# An Effective Solution to Discover Synergistic Drugs for Anti-Cerebral Ischemia from Traditional Chinese Medicinal Formulae

**DOI:** 10.1371/journal.pone.0078902

**Published:** 2013-11-13

**Authors:** Shaojing Li, Chuanhong Wu, Jianxin Chen, Peng Lu, Chang Chen, Meihong Fu, Jing Fang, Jian Gao, Li Zhu, Rixin Liang, Xin Shen, Hongjun Yang

**Affiliations:** 1 Institute of Chinese Materia Medica, China Academy of Chinese Medical Sciences, Beijing, China; 2 Information Center, Beijing University of Chinese Medicine, Beijing, China; 3 Institute of Automation, Chinese Academy of Sciences, Beijing, China; 4 College of Pharmaceutical Science, Hebei University, Baoding, Hebei, China; 5 Jiangxi University of Traditional Chinese Medicine of pharmacy, Jiangxi University of Traditional Chinese Medicine, NanChang, Jiangxi, China; University of Naples Federico II, Italy

## Abstract

Recently, the pharmaceutical industry has shifted to pursuing combination therapies that comprise more than one active ingredient. Interestingly, combination therapies have been used for more than 2500 years in traditional Chinese medicine (TCM). Understanding optimal proportions and synergistic mechanisms of multi-component drugs are critical for developing novel strategies to combat complex diseases. A new multi-objective optimization algorithm based on least angle regression-partial least squares was proposed to construct the predictive model to evaluate the synergistic effect of the three components of a novel combination drug Yi-qi-jie-du formula (YJ), which came from clinical TCM prescription for the treatment of encephalopathy. Optimal proportion of the three components, ginsenosides (G), berberine (B) and jasminoidin (J) was determined via particle swarm optimum. Furthermore, the combination mechanisms were interpreted using PLS VIP and principal components analysis. The results showed that YJ had optimal proportion 3(G): 2(B): 0.5(J), and it yielded synergy in the treatment of rats impaired by middle cerebral artery occlusion induced focal cerebral ischemia. YJ with optimal proportion had good pharmacological effects on acute ischemic stroke. The mechanisms study demonstrated that the combination of G, B and J could exhibit the strongest synergistic effect. J might play an indispensable role in the formula, especially when combined with B for the acute stage of stroke. All these data in this study suggested that in the treatment of acute ischemic stroke, besides restoring blood supply and protecting easily damaged cells in the area of the ischemic penumbra as early as possible, we should pay more attention to the removal of the toxic metabolites at the same time. Mathematical system modeling may be an essential tool for the analysis of the complex pharmacological effects of multi-component drug. The powerful mathematical analysis method could greatly improve the efficiency in finding new combination drug from TCM.

## Introduction

The mainstream international medical community recognizes that single-constituent and single-target drugs are limited. The success of cocktail therapy, which represents multicomponent anti-AIDS drugs, has stimulated people's interest in combination drugs [1]. Combination drugs that simultaneously impact multiple targets are more effective in controlling complex disease systems, such as stroke, than drugs designed to act against individual molecular targets [2]. The complexity of medicine suggests that treatment protocols should be carefully designed; prescription construction in combination drugs is an art to fight disease. Traditional Chinese medicine (TCM) is one of the few rare ancient traditional techniques still widely practiced that holds systematic theories to prevent and therapy diseases. To enhance therapeutic efficacy and reduce adverse effects, practitioners of TCM prescribe a combination of plant species and/or minerals, called formulae, based on their clinical experience. Nearly 100,000 formulae have been recorded; however, the mechanism of action for most remain unknown [3]. It is believed that, at least in some formulae, multiple components affect multiplex targets and exert synergistic therapeutic efficacies. In 2008, Chen et al succeed in explaining the combination mechanisms of Realgar-Indigo naturalis formula, which had been proven to be very effective in treating human acute promyelocytic leukemia [3]. However, the optimal proportions and precise mechanisms of most formulae remain unidentified. Thus, the concerns listed above have hampered the development of TCM. Traditional statistical methods have limitations in the process to deal with complex relationship of formulae. It is urgent to explore new mathematical analysis method for the interpretation of complex combination mechanisms.

Ischemic stroke ranks among the leading causes of death and adult disability worldwide. The pathophysiology of stroke is complex; it involves excitotoxicity mechanisms, inflammatory pathways, oxidative damage, ionic imbalances, apoptosis, angiogenesis and neuroethology injury. In the last several decades, mitochondrial dysfunction has received much attention in the complex pathology of cerebral ischemia [4]. Assays of membrane integrity, membrane fluidity, and mitochondrial membrane potential are commonly used to determine mitochondrial function [5]. Furthermore, mitochondria are pivotal regulators in the process of cell death and survival. Mitochondrial permeability transition (mPT), the release of apoptogenic proteins such as cytochrome c, regulation of apoptosis via proteins in the Bcl-2 family and caspase activation are mechanisms that are involved in neuronal death in which mitochondria constitute the site of action [6], [7].

Ginseng, the root of *Panax ginseng* C. A. Meyer (Araliaceae), has been used as a tonic to treat a wide variety of disorders in traditional Chinese medicine (TCM) for millennia [8]. Rhizoma Coptidis (*Coptis chinensis* Franch, *Coptis deltoidea* C. Y. Cheng et Hsiao, *Coptis teeta* Wall) and Fructus Gardeniae (*Gardenia jasminoides* Ellis) have been used for detoxification [9]–[11]. The clinical effect of TCM formulae containing ginseng, coptis and gardenia was significant. It had been reported that ginsenosides (G) may combat cerebrovascular disease by promoting vasodilation [12], [13], ameliorating cerebral metabolism [14], improving the recovery of neurological functions [15], protecting endothelial cells [16], and increasing the fluidity of mitochondrial membranes [17]. As the active ingredients of coptis and gardenia, berberine (B) and jasminoidin (J), respectively, both have anti-inflammatory and antioxidative effects. They may neutralize toxins and reduce toxic metabolites [18]–[23]. J may reduce the expression of TNF-α, IL-1β [24], inhibit the cytotoxicity [25], lipotoxicity [26]. It has reported that J possesses the potential for detoxication [27]. PI3K/Akt is the anti-apoptosis factors. GSK is the substrate of Akt [28] and NF-κB closely related to inflammation. B may increase the activation of Akt/GSK signaling and claudin-5, and decrease NF-κB expression [29], enhanced PI3K p55γ promoter activity during cerebral ischemia-reperfusion [30]. We prescribed the main bioactive ingredients of the herb mentioned above, namely, G, B and J combined as a formula, called Yi-qi-jie-du formula (YJ), and evaluated its treatment's viability as a new combination drug for acute ischemic stroke.

The analysis of data generated from combined drug researches plays a key role in the process of explaining the action mechanisms of new drugs. Based on the experimental data, it is helpful that developing predictive models to find the optimal proportion of ingredients to determine the synthesized effect of the three components. It may be beneficial to significantly improve the efficiency of the present experiment. Regression analysis is one of the most important methodologies, which is used to construct predictive models, since most pharmacological variables are quantitative. Data analysis methods could be applied to determine the relationship between pharmacological indices and the three YJ components, their impact on pharmacological indices, and combined action mechanisms of the drug. In our experiment, several mathematical methods, including partial least square (PLS), artificial neural network (ANN), particle swarm optimization (PSO) and principal components analysis (PCA), were proposed to analyze and optimize our experiment data.

Understanding optimal drug proportions and synergistic mechanisms of multicomponent drugs are critical for developing novel strategies to cope with complex diseases. It is believed that combinations of agents can effectively reduce side effects and improve adaptive resistance, and in that way, it is able to increase the probability of fighting complex diseases, such as stroke, in a synergistic manner [31]. Mathematical system modeling may be an essential tool for the analysis of the pharmacological effects of multi-component drug to highlight the complex relationship between drugs and their targets, which are characterized as a small sample size and the dispered, nonlinear data, but until now its application is still limited and the successful case is rare in this area. It was prospected that this study would lay foundation to scientific analysis and evaluation of the complex interaction of the multicomponent and further clarify the nature of combination drug via the dissection of YJ in this paper.

## Materials and Methods

### 2.1 Animals

Adult male Sprague–Dawley rats weighing 250–270 g were obtained from the Animal Breeding Center of the Beijing Vital River Laboratories Company (Beijing, China). All animals were individually housed at 22±2°C with a relative humidity of 50±10% and a 12 h light/12 h dark cycle. The animals had free access to food and water. The experimental procedures were approved by the China Academy of Chinese Medical Science's Administrative Panel on Laboratory Animal Care. All animal experiments were performed in accordance with institutional guidelines and ethics.

### 2.2 Chemicals and Reagents

Ginsenosides (Rg_1_+Re+Rd+Rb_2_+Rb_1_+Rb_3_+F_1_+F_2_+Rc+Rg_3_ = 82.62±2.98%, see Table S3, S4, and S5, Figure S1–S26) was purchased from Nanjing ZeLang Medical Technology Co., Ltd (Nanjing, China). The ginsenosides standards for ginsenoside Rb_1_ (Rb_1_), ginsenoside Rb_2_ (Rb_2_), ginsenoside Rb_3_ (Rb_3_), 20(S)-ginsenoside F_1_ (F_1_), 20(S)-ginsenoside F_2_ (F_2_), ginsenoside Rg_1_ (Rg_1_), ginsenoside Rg_3_ (Rg_3_), ginsenoside Rc (Rc), ginsenoside Re (Re), ginsenoside Rd (Rd) were purchased from National Institutes for Food and Drug Control (Beijing, China). Methanol and acetonitrile were purchased from Thermo Fisher Scientific Inc. (Iowa, USA). Berberine (Purity≥95.18%) was purchased from Xianyang Aviation 168 Bio-engineering Co., Ltd (Xianyang, China). Jasminoidin (purity ≥99.68%) was purchased from Baoji F.S. Biological Development Co., Ltd (Baoji, China). EGb761 was purchased from Dr. Willmar Schwabe (Karlsruhe, Germany). Resazurin sodium salt, 2, 3, 5-triphenyltetrazolium chloride (TTC), 1,6-diphenyl-1,3,5-hexatriene (DPH), rhodamine 123, paraformaldehyde, glutaral, osmic acid, propylene oxide, resin, uranyl acetate and lead citrate were purchased from Sigma Chemical Co. (St. Louis, MO, USA). Rabbit polyclonal anti-Bax (P-19) and anti-active caspase-3 antibodies and mouse monoclonal cytochrome c (A-8) and β-actin antibodies were purchased from Santa Cruz Biotechnology (Santa Cruz, CA, USA). Bcl-2 (50E3) was purchased from Cell Signaling Technology (Beverly, MA, USA). The JC-1 (lipophilic cation 5, 5′, 6, 6′-tetrachloro-1, 1′, 3, 3′-tetraethyl-benzimidazol-carb-ocyanine iodide) Mitochondrial Membrane Potential Detection Kit was purchased from the Beyotime Institute of Biotechnology (Haimen, China). The BCA Protein Assay Kit and the Super ECL plus Western Blotting kit were purchased from Life Technologies (Carlsbad, CA, USA).

### 2.3 Animal models and experimental design

After 48 h of acclimatization, rats were anesthetized with chloral hydrate at a dose of 400 mg·kg^−1^ (i.p.). Rectal temperature was recorded and maintained at 37±0.5°C throughout the surgical procedure. The middle cerebral artery occlusion (MCAO) operation by the intraluminal filament method was performed according to a slightly modified previously reported method [32]. Briefly, a 4–0 monofilament nylon suture with a round tip was inserted from the left external carotid artery into the lumen of the internal carotid artery to occlude the origin of the MCA. The rats were sacrificed at 12 h or 24 h after the MCAO procedure.

To dig into the optimal proportions of ingredients in the YJ, the rats were randomly divided into nine groups according to a uniform design (n = 10/group, Table 1). Each group was administered YJ intragastrically (i.g.) 6 h after MCAO; the related indices of mitochondrial function were assayed 24 h after MCAO. To study the anti-cerebral ischemia effect of YJ, rats were randomly divided into the following 6 groups (n = 10/group): sham group; vehicle control group; positive control EGb761 group (4 mg kg^−1^); and YJ-treated groups (1, 5 and 25 mg·kg^−1^). YJ was dissolved in physiological saline to make the stock solution. Dilutions were then prepared for administration of different dosages. YJ and the positive control, EGb761, were administered intragastrically 15 min prior to MCAO (PM) and 6 h after MCAO (AM). The sham and vehicle-treated rats were administered physiological saline intragastrically. Neurological defects were determined at 12 h and 24 h after MCAO followed by an examination of the cerebral infarct volume. The entire brain or cortex was then removed and processed to detect cerebral infarct size, cerebral edema and mitochondrial function. Regional cortical blood perfusion was also determined. Based on the data from the uniform design and pharmacological experiment, a synergistic experimental design (n = 10/group) was proposed to further investigate the mechanisms of the YJ. Each group was administered treatment intragastrically 6 h after MCAO, and the related indices of mitochondrial function were assayed at 24 h after MCAO.

**Table 1:** The uniform design of the YJ.

Group	Ginsenosides (mg·kg^−1^)	Berberine (mg·kg^−1^)	Jasminoidin (mg·kg^−1^)	Dose (mg·kg^−1^)
1	3.273(3)	3.636(7)	1.091(9)	8.000
2	4.909(6)	2.545(4)	1.000(8)	8.455
3	6.545(9)	0.000(1)	0.909(7)	7.455
4	2.727(2)	4.000(8)	0.818(6)	7.545
5	4.364(5)	2.909(5)	0.727(5)	8.000
6	6.000(8)	1.818(2)	0.636(4)	8.636
7	0.000(1)	4.364(9)	0.545(3)	4.909
8	3.818(4)	3.273(6)	0.455(2)	7.545
9	5.455(7)	2.182(3)	0.000(1)	7.636

### 2.4 Uniform experimental design

To explore the optimal proportions of the YJ, a new experimental design method named uniform design was used. This method may potentially overcome the drawbacks of orthogonal designs [33], [34]. This method seeks to design points to be uniformly scattered in the experimental domain [34]. The main difference between the uniform design and the orthogonal design is that the uniform design ensures that there is an experiment is conducted for each factor in each level once only. Consequently, this method can significantly reduce the number of experiments. The uniform design, like the orthogonal design, can be tabulated. It uses a uniform table to organize the factors and levels of each factor. The table is represented as U_n_ (q^s^), where U represents the uniform design, n is the number of experiments, q is the number of levels, and s is the maximum number of factors. The detail of the uniform design of the YJ is shown in Table 1.

For the uniform design, the experimental design process was as follows:

1. Determining the factors and number of levels.
2. Choosing an appropriate table to accommodate the number of factors and levels.
3. Conducting the experiments indicated in the table to collect data on the effect on the performance measure.
4. Completing data analysis to find a suitable model to fit the data.
5. Determining the optimal factor combinations, or discover information built into model.

### 2.5 Preparation of rat brain mitochondria

Rat brain mitochondria were isolated from the left cortical tissue 24 h after the MCAO procedure. Forebrain tissue was quickly removed and placed in ice-cold isolation buffer (250 mM sucrose containing 10 mM Tris-HCl, 0.5 mM Na_2_EDTA and 0.1% BSA, pH 7.1). The tissue was then washed to remove redundant blood and homogenized [20% (w/v)]. Cellular nuclei and cell debris were sedimented by centrifugation at 600 rpm for 3 min and 1000 rpm for 5 min and then discarded. The supernatant was subjected to further centrifugation at 10,000 rpm for 8 min. The mitochondrial pellet was washed by gently resuspending the pellet in isolation medium and then centrifuging at 10,000 rpm for 8 min [35]. Finally, the mitochondria were resuspended in the above buffer to obtain a concentration of 10 mg·ml^−1^. All procedures were performed at 4°C. The mitochondrial protein concentration was determined via BCA assay.

### 2.6 Measurement of mitochondrial viability (Resazurin)

Resazurin is a sensitive indicator of mitochondrial function, which is hydrolyzed to fluorescent resorufin by enzymatic action related to mitochondrial activity [36]. Mitochondria (50 μg protein) were added into a 96-well plate and incubated with 5 μM resazurin at 37°C. Fluorescence intensity was measured after one hour of incubation using a microplate reader (SpectraMax M5, Molecular Devices, Sunnyvale, CA, USA) set to an excitation wavelength of 530 nm and emission wavelength of 590 nm. Samples containing equal amounts of mitochondrial protein that had been heated to 100°C for 10 min prior to the addition of resazurin were used to obtain the background signal. Greater fluorescence intensity of resorufin indicated better resultant mitochondrial viability.

### 2.7 Measurement of mitochondrial swelling (PT)

Mitochondrial swelling following PT pore opening was assayed by measuring the reduction in absorbance at 520 nm at 25°C, according to the method used by Tian [37]. The turbidity of the reaction mixture reflected the degree of mitochondrial swelling. The assay mixture contained freshly prepared mitochondrial protein (50 μg protein), 70 mM sucrose, 10 mM succinate, 5 mM Hepes, 1 mM Na_2_HPO_4_, 210 nM mannitol, 2.7 μM rotenone, and 1 μg·ml^−1^ oligomycin A (pH 7.4). A kinetic decrease in absorbance was recorded over a period of 10 min in 200 μl medium using the microplate reader described above. The control group had the same amount of mitochondria without rotenone and oligomycin A. The detected absorbance had a good linear relationship and the absolute slope was used to compare each group. Steeper slopes indicated greater mitochondrial swelling [38].

### 2.8 Measurement of Mitochondrial Membrane fluidity (FP)

Membrane fluidity was measured by the fluorescence polarization (FP) method using diphenylhexatriene (DPH) as a probe, as previously described by Hirano [39]. DPH (5 µM) was added to freshly prepared mitochondria in medium (250 mM sucrose containing 10 mM Tris-HCl, 0.5 mM Na_2_EDTA and 0.1% BSA, pH 7.1), which were incubated at 37°C for 30 min to allow probe incorporation. The mp value was monitored at 37°C with the microplate reader described above. The excitation wavelength was 362 nm, and the emission wavelength was 432 nm. To identify the effect of YJ, η, which represents the coefficient of viscosity of the mitochondrial membrane, was calculated according to the formula:
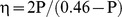
Where the values of P were calculated using the formula P = 1000× mP. Greater values of η indicated reduced fluidity of the mitochondrial membrane.

### 2.9 Measurement of Mitochondria Transmembrane Potential (MMP, Δψ_m_)

#### 2.9.1 Rhodamine 123 Method (Rho)

Changes in brain mitochondrial Δψ_m_ were measured in the presence of rhodamine 123 (Rh123) as described previously [40]. The excitation and emission wavelengths for Rh123 were 503 and 527 nm, respectively, using a SpectraMax M5 Microplate Reader (Molecular Devices, Sunnyvale, CA, USA). Mitochondrial Δψ_m_ was assessed based on the quantitation of Rh123 quenching. Low Δψ_m_ levels corresponded to greater Rh123 fluorescence. The basal fluorescence ([Rh123] total) was determined before adding mitochondria. The mitochondria (0.5 mg protein) were added and incubated in 150 μl buffer (150 mM sucrose, 5 mM MgCl_2_·6H_2_O, 5 mM succinate, 5 mM KH_2_PO_4_, 20 mM Hepes, 2.7 μM rotenone, and 0.5 μM rhodamine 123, PH 7.4) for 1 h. Following the uptake of Rh123, the fluorescence quenching of Rh123 was measured ([Rh123] out). Mitochondrial membrane potential was calculated with the Nernst equation [41].

#### 2.9.2 JC-1 Method (JC1)

As another method, the JC-1 Mitochondrial Membrane Kit (Beyotime Institute of Biotechnology, Haimen, China) was also used to monitor changes in Δψ_m_. JC-1 is an ideal fluorescent probe for the detection of Δψ_m_. When Δψ_m_ is high, JC-1 aggregates in the matrix of the mitochondria, producing red fluorescence. By contrast, when Δψ_m_ is low, JC-1 exists in its monomeric form and produces green fluorescence. The ratio of the red to green fluorescence intensity at 590 nm compared to that at 530 nm was used to measure depolarization of the mitochondrial membrane [42].

### 2.10 Data Analysis

#### 2.10.1 Data Preprocessing

It was necessary that some data preprocessing steps were prior to analysis to reduce or eliminate any outliers, missing values, or bad data points, and ensure that the data were perfectly suitable for modeling [43], [44]. Normalization procedures were applied to meet these challenges for integrated data analysis. All of the data of mitochondrial function from the uniform design experiment was divided by two groups: a factor group and an indexed group. The factor group had 9 rows and 3 columns (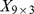), and the indexed group had 9 rows and 5 columns (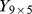). Each group was transformed the mean and standard deviation of each column to 0 and 1. The mapping function was as follows:
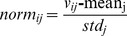
Where 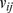 was the original value of 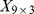 or 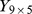, 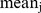 was the mean of column 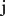, and 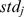 denoted the standard deviation of column 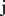.

#### 2.10.2 LARS-PLS

Multi-target regression was a common issue in regression analyses [45]. In cases where samples are small, namely in the fields of medicine, the military field and chemical processes [46], the prediction becomes difficult. Some popular methods [47] do not attempt to fit data in small sample cases. Here, we proposed a method, named by Least Angle Regression-Partial Least Squares to model these cases.

This method included two main parts: a Least Angle Regression algorithm [48] and a Partial Least Squares algorithm [49]. The Partial Least Squares algorithm applied well to small sample cases, while the Least Angle Regression algorithm added non-linear factors and improved fitting accuracy. This method may copy with two major problems in small sample cases. The first problem referred to multicollinearity in the independent variables [50]; the second problem relating to the number of samples was less than the number of variables [51].

Next, we introduced this procedure to one medical case. In this case, we needed to fit the regression model between drug components and efficacy indices. The procedure was illustrated as follows:

1. Choosing drug components as the independent variables and efficacy indices as the dependent variables, according to the actual medical situation.
2. Setting ratios of the drug components in experiments according to principles of uniform design. Recording the efficacy indices of each experiment.
3. Assigning the experimental data to independent variables and dependent variables, and forming the independent variable and dependent variable matrices. In the independent matrix, each row corresponds to the component ratio in each drug experiment, and each column corresponds to one drug component. In the dependent matrix, each row corresponds to each efficacy index of drug experiment, and each column corresponds to one efficacy index.
4. Expanding the independent matrix to add nonlinear factors. As to each row, calculate the quadratic value between each component index in turn. Add the quadratic values of each sample to the end of corresponding row. In this way, the new independent matrix is established.
5. Applying the Partial Least Squares algorithm to retrieve principle components. Firstly, set the number of principle components as ncomp, and retrieve useful information from the new independent matrix through projecting the independent matrix to the scoring matrix, in which the number of rows is the number of samples and the number of columns is ncomp. Similarly, project the dependent matrix to the scoring matrix. Build the PLS regression model between these two scoring matrices.
6. Finally, projecting the principle components to the original variables in the reverse direction and obtain the regression model between drug components and efficacy indices.

Following these procedures, we were able to produce regression models for multiple targets in small sample cases. Moreover, given a set of drug components, this model could be used to predict efficacy indices.

To seek the optimal proportion of YJ, particle swarm optimization (PSO) was proposed. PSO has become a popular evolutionary algorithm in recent years, based on stochastic optimization techniques developed by Dr. Eberhart and Dr. Kennedy in 1995, which were inspired by social behavior research of bird flocking or fish schooling [52], [53].

In PSO, the system is initialized with a swarm of random solutions, called particles in the problem space, and the system searches for optima by updating generations. Each of the particles refers to a fitness value associated with the optimal function and velocity determining the direction and distance of each particle. It also keeps track of the coordinates of the current best solution previously achieved. During each time step, the particle is updated by tracking two extreme values. One, called the personal-best (pbest), is the best solution the current particle has achieved so far. The other, called the global-best (gbest), is the best solution the entire swarm has encountered so far, which is continually updated by comparison with pbest values. If a current particle has reached a better optimization location, gbest will be updated and the next particle in the swarm will try to make its way towards the gbest location. In past several years, PSO has been successfully applied to many areas of research and application [44]. PSO has also been demonstrated to obtain better results in a method that is both faster and cheaper than other methods.

### 2.11 Pharmacological effects study for YJ under the optimal proportion

#### 2.11.1 Assessment of neurological defects

To reveal the effect of YJ the neurological defects caused by the MCAO operation, neurological defects were determined by a single researcher at 12 h and 24 h after MCAO. The researcher was blinded to the experimental treatment groups. The neurological behaviors were scored on the following 5-point scale as described previously [54].

#### 2.11.2 Cerebral infarct size

Cerebral infarct volumes were measured with TTC staining and used to describe the severity of cerebral ischemia. At 12 h and 24 h of ischemia, brains were quickly removed and sliced into 6 2-mm thick coronal sections. Brain slices were treated with 2% TTC saline solution and incubated at 37.5°C for 30 min, followed by 10% formalin fixation overnight, according to a previously described method [55]. After staining with TTC, normal tissue was stained a rose red color, and the infarct tissue was stained white. The images of the stained slices were photographed and recorded. The adjusted infarct areas and bilateral hemispheric areas of each slice were determined using an image analysis system (Image-pro plus 6.0). The infarct volume of each slice was calculated as the infarct area × thickness (2 mm). The summation of the infarct volumes of all brain slices was recorded as the total infarct volume.

#### 2.11.3 Evaluation of cerebral edema

Following decapitation at 12 h and 24 h after MCAO, the left cerebral hemispheres were obtained as described above and immediately weighed to obtain the wet weight. The tissue was then dried in an oven at 120°C for 24 h and then reweighed to obtain the dry weight. Cerebral water content [56] was calculated according to the following formula:

Content of cerebral water  =  [(wet weight – dry weight)/wet weight] ×100%.

#### 2.11.4 Regional cortical blood perfusion

**Figure 1:**
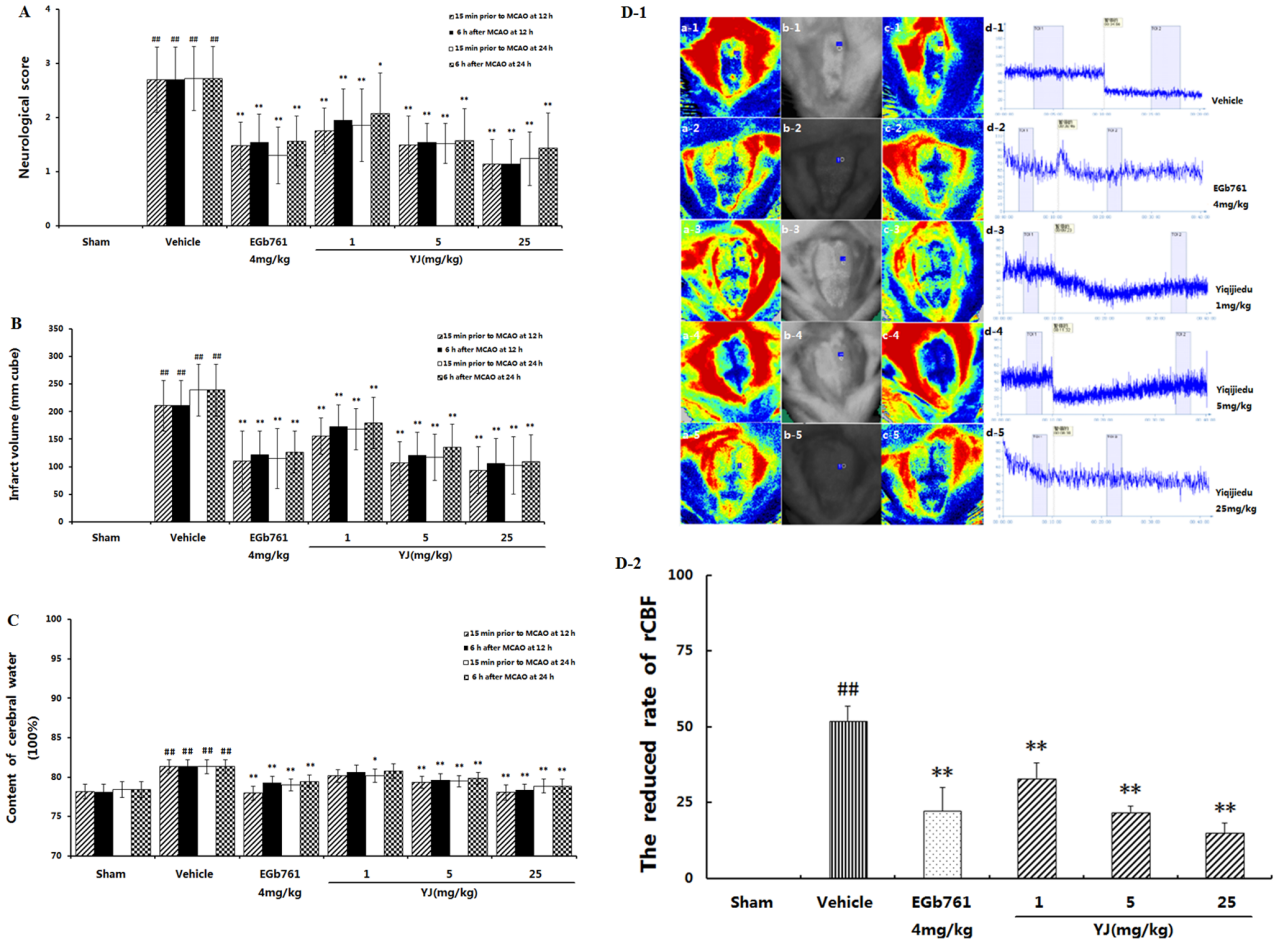
The therapeutic effects of YJ with the optimal proportion. (A)–(C) Effects of YJ on neurological deficits, cerebral infarct volume and content of cerebral water in rats induced by MCAO. EGb761 and YJ were both administered i.g. 15 min prior to MCAO and 6 h after MCAO. (D-1) Detection of cerebral blood flow. The cerebral blood flow perfusion color image [D-1. a (1–5) and c (1–5)], detected position image [D-1. b (1–5)], and blood flow time window [D-1. d (1–5)] are presented. [1, Vehicle control; 2, EGb761-treated group; 3–5, YJ-(1,5 and 25 mg·kg^−1^) treated group]. (D-2) The reduced rate of rCBF. EGb761 and YJ were both administered i.g. 15 min prior to MCAO. Values were expressed as the mean ± SD (A, B and C, n = 10; D, n = 5), and the data were analyzed by one-way ANOVA. ^##^ *P*<0.01,^#^ *P*<0.05 versus the sham group; ^**^ *P*<0.01,^*^ *P*<0.05 versus the vehicle control.

Laser speckle contrast imaging (LSCI) is a technique based on speckle contrast analysis that provides an index of blood flow [57]–[59]. A reduction in rCBF plays an essential role in ischemia-induced brain injury. To evaluate the effect of YJ, rCBF was measured before and after MCAO for each group. After deep anesthesia, each rat experienced a skin incision to expose the skull in a supine position prior to the test. The probe was positioned 10 cm above the detected frontoparietal cortex region of the left hemisphere. A round window of approximately 0.8 mm^2^ in size had been located below and to the left of the bregma, just adjacent to the MCA area. The cerebral blood flow perfusion color image (Figure 1D-1. a and c), detected position image Figure 1D-1.b), and blood flow time window (Figure 1D-1.d) were recorded.
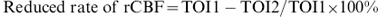



Where the value of TOI1 and TOI2 were calculated as a percentage of the baseline value 15 min prior to and 30 min after the MCAO, respectively. YJ and EGb761 were administered intragastrically 15 min prior to MCAO.

#### 2.11.5 Western blot analysis for apoptosis related protein-Cortex proteins

The rat brain homogenate in ice-cold lysis buffer containing 150 mM NaCl, 25 mM Tris–HCl, 1 mM EGTA, 1 mM EDTA, 1% Triton X-100, 0.5% NP-40, 1 μg·ml^−1^ aprotinin, 1 μg·ml^−1^ leupeptin, and 1 mM PMSF, pH 7.4, was used for Western blot experiments [20%(w/v)]. The homogenate was incubated on ice for 30 min and then centrifuged at 12,000 rpm for 20 min at 4°C. The supernatant was collected, and the protein concentrations of the extracts were measured by BCA assay. The protein samples in the supernatant were resolved by SDS-polyacrylamide gel electrophoresis (SDS-PAGE) and electrotransferred to PVDF membranes [41]. The membrane was incubated with the respective primary antibodies against Bcl-2 (1∶1000), Bax (1∶1000) and activated caspase-3 (1∶1000) overnight at 4°C. The antibody for β-actin (1∶5000) served as the loading control. Finally, the membrane was incubated with horseradish peroxidase-conjugated secondary antibody. Protein bands were visualized using the ECL Western blotting detection kit. The relative intensities of the bands were quantified by densitometric analysis. The densitometric plots of the results were normalized to the intensity of the actin band.

#### 2.11.6 Western blot analysis for apoptosis related protein-Mitochondrial proteins

The release of mitochondrial cytochrome c was determined by Western blot experiments according to the method described by He [41]. The rat brain homogenate in ice-cold lysis buffer mentioned above [20% (w/v)] was centrifuged at 1000 rpm for 10 min, and the resulting supernatant was centrifuged at 10,000 rpm for 10 min. The pellet contained the mitochondrial fraction. The supernatant was then re-centrifuged at 10,000 rpm for 1 h at 4°C. The resulting supernatant was used as the cytosolic fraction. The forty microgram proteins in pellet and supernatant were prepared and immune blotted with cytochrome c antibody (1∶1000). The relative intensities of the bands were also quantified by densitometric analysis. The densitometric plots of the results were normalized to the intensity of the actin band.

#### 2.11.7 Morphological studies of mitochondria and brain tissue cells

Transmission electron microscopy was used to observe the ultrastructural changes of mitochondria and brain tissue cells. The sham, vehicle and YJ-treated (25 mg·kg^−1^) group rats were anesthetized, perfused with 0.9% NaCl (1000 ml·kg^−1^), and then reperfused with 4% paraformaldehyde (1000 ml·kg^−1^) at 24 h after reperfusion. The parietal cortex was cut into 1 mm^3^, fixed with 2.5% glutaral for 2 h, and washed three times with PBS, 10 min once. Then the tissue was fixed with 1% osmic acid for 2 h, sequentially, washed with pure water, dehydrated with ethanol, replaced with propylene oxide and resin mixture, embedded in pure resin, and stained with uranyl acetate and lead citrate [60]. The cortical morphology was observed under H-7650 transmission electron microscope (Hitachi, Japan) and photographed.

#### 2.11.8 Statistical analyses

The data were expressed as the mean ± SD. The statistical significance of the differences between groups was determined by one-way analysis of variance (ANOVA). *P* value <0.05 was considered statistically significant.

### 2.12 The study of the combination mechanisms of YJ

#### 2.12.1 Measures of variable importance to indices

The main goal of this portion of the present study was to identify the influence of YJ on five indices that explain changes in the experimental data. In this work, the methodology used was the calculation of variable importance in the project (partial least squares algorithms) scores. The VIP statistic represents the influence on the *Y*-responses of every predictor *X* value in the model. In fact, the VIP values reflect the importance of independent invariables in the predictive model with respect to Y.

In this paper, the LARS-PLS model was developed to describe the quantitative relationship between the independent variables (X) and the response variables (Y). Based on the LARS-PLS model, the influence of the predictors by means of their importance in the prediction was investigated, according to the VIP score. The VIP score for the i^th^ variable was calculated as [61]:
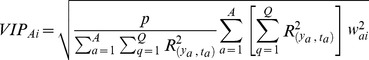
Where 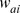 was the loading weight for variable 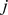 using component 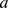.

#### 2.12.2 Synergistic experimental design

The formulae of TCM exhibited complex characteristics, which gave rise to difficulties in identifying the synergistic effect of each component. Based on the pharmacological experiment and uniform design experiment, we designed an experiment to prove whether the three components used together would demonstrate the maximum pharmacological effect.

In this experiment, two doses were used: 5 mg·kg^−1^ and 25 mg kg^−1^. In each dose, three components, only one component and only two components were used, respectively. Fourteen experiments were conducted More details are shown in Table 2.

**Table 2:** The synergistic experimental design of the YJ.

Group	Ginsenosides (mg·kg^−1^)	Berberine (mg·kg^−1^)	Jasminoidin (mg·kg^−1^)	Dose (mg·kg^−1^)
1	13.64	9.09	2.27	25
2	25	0	0	25
3	0	25	0	25
4	0	0	25	25
5	12.5	12.5	0	25
6	0	12.5	12.5	25
7	12.5	0	12.5	25
8	2.73	1.82	0.45	5
9	5	0	0	5
10	0	5	0	5
11	0	0	5	5
12	2.5	2.5	0	5
13	0	2.5	2.5	5
14	2.5	0	2.5	5

## Results

### 3.1 Multi-objective predictive models and optimal proportion of YJ on anti-cerebral ischemia efficacy

To better seek the mechanisms of action of the YJ, the quantitative data of several key indices about mitochondrial function were processed and integrated by data mining methods.

The multi-objective predictive analysis was performed by 3 factors and 5 indices as input variables for the uniform experimental data. We tried to fit the regression model based on the previous obtained nine samples. In total, four algorithms were used to build the computational model between drug components and medical indices, namely linear partial least squares (PLS) regression, multi-linear regression, Back-Propagation neural network regression (BPNN) and least angle regression-partial least squares (LARS-PLS) regression. Moreover, several indices were selected to evaluate the performance of each model to find the most suitable model for estimating target values.

**Figure 2:**
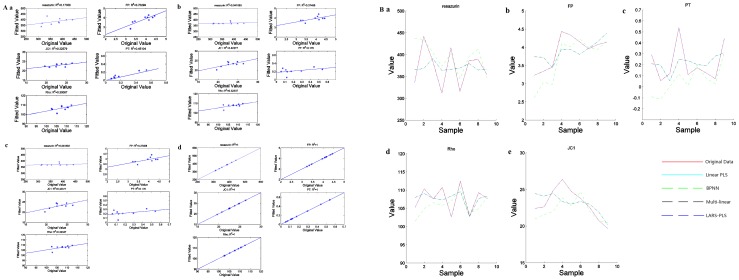
The construction of the predictive model for YJ. A. The values of R^2^ for the five medical indices according to different calculation models; (A-a to d) The values of R^2^ for the BPNN regression model, linear PLS regression model, multi-linear regression model and LARS-PLS regression model. B. Predicted values of the five medical indices for the different models. (B-a to e) Predicted values of resazurin, FP, PT, Rho and JC1 for different models.

Firstly, we compared the fitted value to the original data. As for each model, straight lines were plotted for the five medical indices to reveal the goodness of fitted values to the original data. The value of R^2^ was computed for each line, which measured fitting accuracy. In general, the value of R^2^ became closer to 1 when the points in the scatter plot moved closer to the straight line, indicating that the model fitted the original data more accurately. In cases of a perfect fit, all points fall on a straight line, and R^2^ = 1 accordingly. The values of R^2^ for the five medical indices were computed according to different computational models, shown in Figure 2A.

Through this comparison, it had been observed that the LARS-PLS regression model had the best performance among the four models. Considering that the number of samples was small, a slight change in the samples would affect the position of the plotted line. Thus, we sought other indices for further evaluation.

We plotted the predicted values of the four models on the same figure and compared their closeness with the original data. As was shown in Figure 2B, the four models achieved different performance in the five medical indices. Obviously, models, fitting the data more accurately, were more likely to decipher the inner mechanisms between the drug components and medical indices.

Based on these figures, we used two indices, namely the THILE Index and RMSE, to quantify the performance of the four models. The THILE Index was computed as
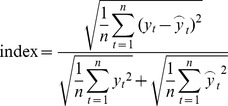
where y_t_ was the original medical value and 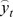 was the corresponding predicted value. Smaller THILE Index values indicated more accurate predictions; a perfect predictor's THILE Index  = 0. Considering that BPNN gave different predictions in different trials, we repeated it for ten times and took the average value as the performance of the neural network regression model (see Table S1).

As illustrated in the Supporting Information (Table S2), LARS-PLS was again demonstrated to obtain the best performance among the four models. The LARS-PLS model predicted the five target indices with the smallest THILE Index and RMSE values. The superiority of the LARS-PLS model could be divided to two aspects: the PLS algorithm had been validated to perform well in small sample cases, and LARS had been shown to improve the accuracy of the model by adding non-linear factors.

Thus, we chose the LARS-PLS model as the best predictor of the medical indices. Combined with the particle swarm optimization algorithm, the LARS-PLS model was used to find the optimal composition of the YJ. After searching the composition space using PSO, we sought the local optimal composition, illustrated in Table 3. The optimal proportion of the three components was approximately 3∶2∶0.5. The data, shown in Table 3, indicated that when the three drug components were given in this ratio, the five medical indices would be 429.8514, 4.0134, 24.6169, 0.1190, 104.0423 as the LARS-PLS prediction, respectively.

**Table 3:** Optimal composition and target values.

Ginsenosides	Berberine	Jasminoidin	Resuzurin	FP	JC1	PT	Rho
4.4205	2.9546	0.8669	429.8514	4.0134	24.6169	0.1190	104.0423

### 3.2 Validation of the optimal proportion of mitochondrial function

#### 3.2.1 Measurement of mitochondrial vitality (Resazurin)

Mitochondrial function was evaluated by measuring the fluorescence intensity of resorufin. As shown in Table 4, the mitochondrial viability was significantly reduced in the vehicle-treated group. This value decreased from 531.33±152.53 to 330.28±61.42 as a result of MCAO (*P*<0.01). With the exception of the low-dose YJ group, the YJ-treated groups at doses of 5 mg·kg^−1^ and 25 mg·kg^−1^ and the EGb761-treated group at a dose of 4 mg·kg^−1^ experienced greater improvements in mitochondrial function (*P*<0.01) than the vehicle control.

**Table 4:** The mitochondrial function index of the verification experiment.

Group	Dose (mg·kg^−1^)	n	Reaszurin	PT	FP	MMP	
						Rho	JC1
Sham	-	10	531.33 ±152.53	0.152±0.012	3.67±0.30	131.53±2.23	26.58±1.49
Vehicle	-	10	330.28±61.42 ^##^	0.678±0.011^##^	5.89±3.11^##^	125.17±4.70^##^	21.80±3.44^##^
EGb761	4	10	427.20±87.05 ^**^	0.276±0.021^**^	3.70±0.49^**^	129.92±3.98^*^	24.68±2.69^*^
YJ	1	10	377.02±109.08	0.269±0.010^**^	4.03±0.47^**^	127.05±2.56	22.01±2.03
YJ	5	10	440.92±75.89^**^	0.164±0.008^**^	3.74±0.55^**^	130.20±3.04^**^	21.88±0.84
YJ	25	10	481.91±69.99^**^	0.159±0.007^**^	3.49±0.51^**^	129.44±2.41^*^	24.67±1.94^*^

Values were expressed as the means ± SD (n = 10) and the data were analyzed by one-way ANOVA.^ ##^ *P*<0.01, ^#^ *P*<0.05 versus the sham group; **P<0.01, *P<0.05 versus the vehicle control.

#### 3.2.2 Measurement of mitochondrial swelling (PT)

Reduced OD at 520 nm indicated mitochondrial swelling. Mitochondrial swelling was more serious in the vehicle control compared to the sham group. The group treated with EGb761 and YJ (1 mg·kg^−1^, 5 mg·kg^−1^ and 25 mg·kg^−1^) showed significantly (*P*<0.01) reduced amounts of mitochondrial swelling (Table 4).

#### 3.2.3 Mitochondrial membrane fluidity measurement (FP)

Mitochondrial membrane fluidity was reflected by membrane viscosity (η). A greater η value indicated reduced fluidity of the mitochondrial membrane. As shown in Table 4, the vehicle-treated group had greater η values (5.89±3.11) compared to the sham group (*P*<0.01). The η values in the groups treated with EGb761 and YJ (1 mg·kg^−1^, 5 mg·kg^−1^ and 25 mg·kg^−1^) were significantly less (*P*<0.01) than that of the vehicle-treated group.

#### 3.2.4 Measurement of Mitochondrial Transmembrane Potential (MMP, Δψ_m_)-The method of rhodamine 123 (Rho)

Changes in Δψ_m_ were monitored by measuring the release of rhodamine 123, which had been preloaded into mitochondria. As shown in Table 4, the Δψ_m_ were significantly decreased from 131.53±2.23 mv to 125.17±4.70 mv as a result of the MCAO (*P*<0.01). The EGb761-treated groups showed an enhanced membrane potential compared to the vehicle-treated group (*P*<0.05). The YJ-treated groups showed an improved membrane potential (*P*<0.05) at a dose of 25 mg·kg^−1^. Treatment with YJ (5 mg·kg^−1^) also led to improved membrane potential (*P*<0.01). However, YJ at a dose of 1 mg·kg^−1^ did not significantly improve the reduction in mitochondrial membrane potential.

#### 3.2.5 Measurement of Mitochondrial Transmembrane Potential (MMP, Δψ_m_)-The method of JC-1 (JC1)

To measure the depolarization of the mitochondrial membrane, the mitochondrial membrane potential was measured with the JC-1 probe. The red/green fluorescence ratio of JC-1 was shown in Table 4. The vehicle-treated group had a significantly smaller ratio than the shame group; the ratio in the vehicle-treated group was reduced from 26.58±1.49 to 21.80±3.44 (*P*<0.01). The EGb761-treated group showed a significantly increased ratio (24.68±2.69; *P*<0.01) compared to the vehicle-treated group. At a dose of 25 mg·kg^−1^, YJ increased this ratio significantly (*P*<0.05); however, YJ at 1 mg·kg^−1^ and 5 mg·kg^−1^ did not significantly increase the ratio.

In conclusion, the YJ of the optimal proportions favored mitochondrial function at 24 h after MCAO. This suggested that the LARS-PLS regression model combined with PSO was relatively accurate in searching for the best ratio of components in the YJ.

### 3.3 The therapeutic effect of YJ with the optimal proportion

#### 3.3.1 Neurological defects

Middle cerebral artery occlusion was performed on the left side, and 12 h/24 h following occlusion, right hind paresis was observed in rats compared to the contralateral side, as shown in Figure 1A. The mean neurological scores in the vehicle-treated groups were significantly (*P*<0.01) higher than the sham groups, indicating neurological defects after the MCAO both at 12 h and 24 h. In the EGb761-treated group and the YJ- (1 mg·kg^−1^, 5 mg·kg^−1^ and 25 mg·kg^−1^) treated groups, the neurological deficits were significantly improved (*P*<0.01 or *P*<0.05, at PM and AM) when compared to the vehicle-treated group at both 12 h and 24 h after MCAO.

#### 3.3.2 Cerebral infarct size

At 12 h and 24 h after MCAO, the mean infarct volumes in the vehicle-treated group were 210.95±45.83 mm^3^ (*P*<0.01) and 239.06±46.72 mm^3^ (*P*<0.01), respectively (Figure 1B). Oral administration of EGb761 and YJ (1 mg·kg^−1^, 5 mg·kg^−1^, and 25 mg·kg^−1^) significantly reduced infarct volume (*P*<0.01) in these groups compared to the vehicle control group both at AM and PM.

#### 3.3.3 Cerebral edema

Middle cerebral artery occlusion was performed on the left side, and the left brain was processed to evaluate the cerebral edema 12 h and 24 h after the occlusion. As shown in Figure 1C, at 12 h and 24 h after MCAO, the content of cerebral water in the vehicle-treated group (81.35±0.87, 12 h; 81.31±0.86, 24 h) was significantly (*P*<0.01) higher that of the sham group. In the EGb761-treated groups, the content of cerebral water was significantly reduced (*P*<0.01) compared to the vehicle-treated group. At 24 h after MCAO, YJ at the 5 mg·kg^−1^ and 25 mg·kg^−1^ dosages improved cerebral edema significantly (*P*<0.01), both at AM and PM, compared to the vehicle-treated group. YJ at the dose of 1 mg·kg^−1^, could reduce the content of cerebral water at PM (*P*<0.05). At 12 h after MCAO, YJ at the 5 mg·kg^−1^ and 25 mg·kg^−1^ dosages improved cerebral edema significantly (*P*<0.01). However, the YJ- (1 mg·kg^−1^) treated group experienced no improvement in cerebral edema when compared to the vehicle-treated MCAO group.

#### 3.3.4. Regional cortical blood perfusion

We detected the cerebral blood flow with using a Perfusion Speckle Imager (PERIMED, Sweden), and the picture is presented in Figure 1D-1. The reduction of cerebral blood flow following MCAO in the vehicle-treated group was (51.95±4.71)% compared to the sham group (*P*<0.01) (Figure 1D-2). The cerebral blood flows of the EGb761- and YJ- (1 mg·kg^−1^, 5 mg·kg^−1^ and 25 mg·kg^−1^) treated groups were significantly enhanced (*P*<0.01) compared to the vehicle-treated group after MCAO.

#### 3.3.5 Evaluation of the Anti-apoptotic effect-Modulation of Bcl-2 and Bax Expression

**Figure 3:**
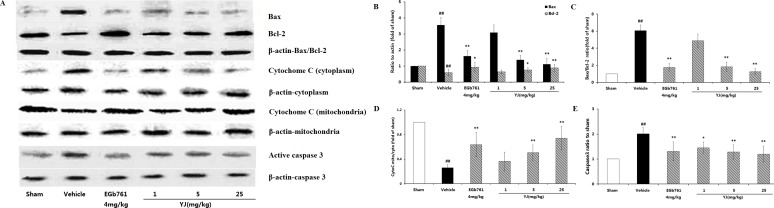
Effects of the anti-apoptotic effect of YJ with the optimal proportion. (A). Representative Western blots of Bcl-2, Bax, Cytochrome c, caspase-3 and β-actin. (B), (D), (E), The quantified densitometric analysis of Bcl-2, Bax, cytochrome c and caspase-3. (C). The ratio of Bax/Bcl-2 proteins. Values were expressed as the mean ± SD for the four independent experiments, and the data were analyzed by one-way ANOVA. ^##^ *P*<0.01,^#^ *P*<0.05 versus the sham group; ^**^ *P*<0.01,^*^ *P*<0.05 versus the vehicle control.

As shown in Figure 3B, Bcl-2, a key protein that contributes to cell survival, was present in relatively high levels in the sham group; it was decreased in the vehicle control at 24 h after the MCAO. In contrast, the level of Bax, an important pro-apoptotic protein, increased markedly in the vehicle control. As shown in Figure 3C, the ratio of Bax/Bcl-2 in the vehicle control increased significantly (6.05-fold of sham); this may be involved in the apoptotic cell death caused by MCAO. In the dose range of 1–25 mg·kg^−1^, YJ reduced the up-regulation of Bax and increased the level of Bcl-2 when administered 6 h after MCAO. Therefore, treatment with YJ was effective in maintaining the balance between Bcl-2 and Bax.

#### 3.3.6 Evaluation of the Anti-apoptotic effect-Modulation of Cytochome c Expression

One of the mechanisms by which Bcl-2 blocks apoptosis is by decreasing cytochrome c release from mitochondria. As shown in Figure 3D, significant translocations of cytochrome c was detected in the vehicle control group, in which the ratio of cytochrome c content in the mitochondrial and cytosolic fractions was approximately 0.26-fold of the sham group (*P*<0.01) at 24 h after MCAO. YJ treatment markedly increased this ratio (*P*<0.01) (Figure 3D) when administered 6 h after the MCAO. These results suggested that YJ could significantly inhibit the release of cytochrome c.

#### 3.3.7 Evaluation of the Anti-apoptotic effect-Modulation of activated Caspase-3 Expression

Caspase-3 is an important executioner in apoptosis because it hydrolyzes a number of structural and signaling proteins involved in this process. As illustrated in Figure 3E, Western blot analysis showed that the level of activated caspase-3 increased significantly after MCAO. However, there was a reduction of increased caspase-3 activation in the YJ- (1 mg·kg^−1^, 5 mg kg^−1^ and 25 mg·kg^−1^) treated groups when YJ was administered 6 h after MCAO. The action of YJ with respect to these molecular events was most likely paralleled YJ's effect on apoptosis [62].

#### 3.3.8 Morphological studies of mitochondria and brain tissue cells

**Figure 4:**
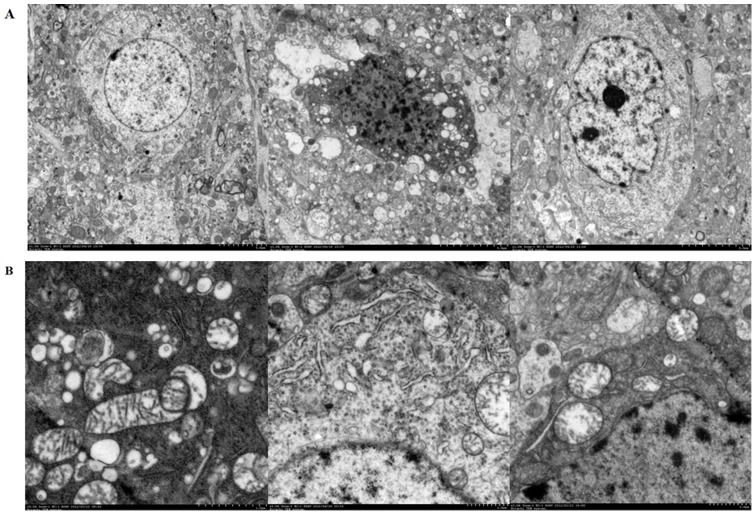
Morphological studies of mitochondria and brain tissue cells. (A) The neurons picture (1000×). (B) The mitochondrial picture (3000×). a. The sham-treated group; b. The vehicle-treated group; c. The YJ-treated (25 mg·kg^−1^) group.

Morphological studies of mitochondria and brain tissue cells were illustrated in Figure 4. As shown in Figure 4A, neurons in sham-treated group had integrated structures. However, the nucleus of neurons was pyknosis and the organelles such as mitochondria were obviously damaged in the vehicle-treated group. Compared with the vehicle-treated group, the cellular morphology and the contents seemed to be improved in the YJ-treated (25 mg·kg^−1^) group. Mitochondrial morphologies were shown in Figure 4B. The mitochondrial cristae appeared tubular with regular intercristal cross-section and cristae junctions in the sham-treated group. Differently, the mitochondria in the vehicle-treated group were swelling in majority, the cristaes were severely damaged and even dissolved. However, in the YJ-treated (25 mg·kg^−1^) group, the mitochondrial swelling was significantly reduced and the structure damage was significantly improved.

### 3.4 The study of the combination mechanisms of YJ

Considering the positive pharmacological effects of the YJ in optimal proportions, we made use of the VIP and synergistic experimental design to the further study of potential mechanisms of action of YJ.

#### 3.4.1 Analysis of the importance of three compounds

**Figure 5:**
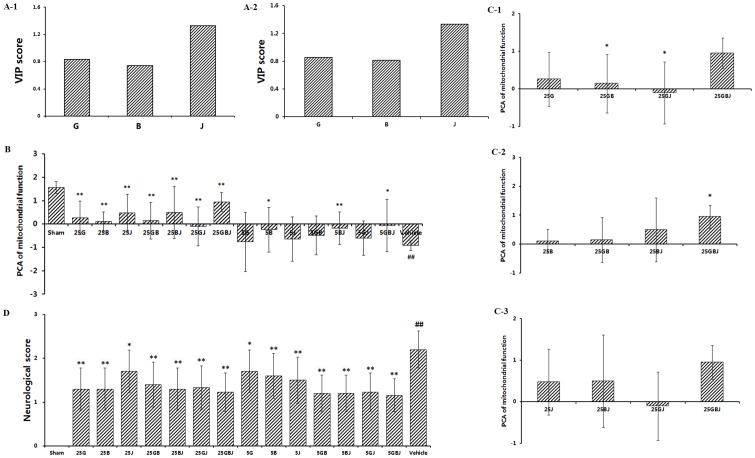
The study of the combination mechanisms of YJ. (A-1) VIP score on linear PLS regression model. (A-2) VIP score on LARS-PLS regression model. (B). The principal component of mitochondrial function. (D). Effects of YJ on neurological deficits induced by MCAO. Values were expressed as the mean ± SD (n = 10), and the data were analyzed by one-way ANOVA.^ ##^ *P*<0.01,^#^ *P*<0.05 versus the sham group; ^**^ *P*<0.01,^*^ *P*<0.05 versus the vehicle control. (C). The analysis of synergy using principal components analysis (PCA) for mitochondrial function. (C-1) The principal components of group G, GB, GJ and GBJ.^ **^ *P*<0.01,^*^ *P*<0.05 versus the G-treated group. (C-2) The principal components of group B, GB, BJ and GBJ. ^**^ *P*<0.01,^*^ *P*<0.05 versus the B-treated group. (C-3) The principal components of group J, BJ, GJ and GBJ. ^**^ *P*<0.01,^*^ *P*<0.05 versus the J-treated group.

VIP was used to measure the importance of each variable to interpret the variables. From the definitional formula of VIP, it is known that VIP is based on the PLSR model. In this paper, VIPs were computed by two PLSR models: the linear PLS regression model and the LARS-PLS regression model. These models were used to analyze the data from the uniform design study to obtain the importance of each of the three components. The linear PLS VIP of the three components is shown in Figure 5A-1. In addition, the LARS-PLS VIP was took into account. Based on Lars, nine variables were generated: X1, X2, X3, X1*X1, X1, X1*X2, X2*X2, X1*X3, X2*X3, and X3*X3. The detailed VIP scores were shown in Figure 5A-2.

According to the results, showed in the linear PLS regression model and the LARS-PLS regression model, the VIP score indicated that J was more important than the other two components, and G was more important than B, which suggested that in the acute stage of MCAO, J played the most important role, even though it was just the component of the least concentration in the YJ.

#### 3.4.2 Principal components analysis (PCA) to expose synergy effect of YJ

Principal components analysis (PCA) was used to analyze mitochondrial function to investigate the integrated effects of G, B and J. We extracted the principle component of the five mitochondrial indices. One-way ANOVA was used to analyze group differences. The principal component was illustrated in Figure 5B. The data from the vehicle-treated group (−0.91±0.20) was significantly (*P*<0.01) less than that of the sham group, indicating greater mitochondrial dysfunction after MCAO. At doses of 5 mg·kg^−1^ and 25 mg·kg^−1^, the data of the GBJ group was significantly greater (*P*<0.05 and *P*<0.01, respectively) than that of vehicle-treated group. The data was also greater than the same dose of mono- or bi-therapy of G, B and J. The GBJ formulation showed strong synergy in preventing mitochondrial dysfunction at 24 h after MCAO. It was interesting that both at the doses of 5 mg·kg^−1^ and 25 mg·kg^−1^, BJ-treated groups had higher scores than the vehicle-treated group (*P*<0.01). The scores of GB- and GJ-treated groups at doses of 5 mg·kg^−1^ were not remarkably different than that of the vehicle-treated group. This suggested that at the acute stage of ischemic stroke, B and J played important roles in treatment.

Considering that at the 5 mg·kg^−1^ dose the difference between drug-treated groups and vehicle-treated groups was not remarkable, 25 mg·kg^−1^ treated group was used to conduct further analyses. As was shown in Figure 5 (C-1, C-2 and C-3), the synergy of GBJ was the strongest in our experiment. When G and J were added to B, the effect of B improved. It was interesting that J could improve the effect of B more than G, suggesting that a combination of B and J was better than any other bi-therapy. When G and B were added to J, they appeared different functions. B could improve the effect of J, but when G was combined with J, the interaction between G and J was reduced. The results showed that the synergy of GBJ was stronger than the interaction of G and B, G and J, and B and J. Among GB-, GJ- and BJ-treated groups, BJ performed the best.

#### 3.4.3 Neurological defects verify the synergistic effect of YJ

As shown in Figure 5D, the mean neurological score in the vehicle-treated group (2.20±0.42) was significantly (*P*<0.01) higher than that of the sham group, indicating the presence of a neurological defect after MCAO. At the doses of 5 mg·kg^−1^ and 25 mg·kg^−1^, the scores of GBJ group were significantly lower (*P*<0.01) than those of vehicle-treated group and groups receiving other treatment as the same dosage. It was interesting that at both the doses of 5 mg·kg^−1^ and 25 mg·kg^−1^, the BJ-treated groups obtained a lower score than the vehicle-treated group (*P*<0.01). This was a result of the synergistic effect on mitochondrial function. It suggested that G, B and J were all indispensable components of the YJ, as indicated by synergy in the SD rats induced by MCAO.

## Discussion

Recently, with the understanding of complex diseases growing, the focus of drug discovery research has shifted from the originally well-accepted “one target, one drug” model to a newer “multi-target, multi-drug” model that aims to systemically modulate multiple targets. The combination of multiple drugs is thought to maximize therapeutic efficacy by facilitating synergistic actions and ameliorating or preventing potential adverse effects, while at the same time, affecting multiple targets [63]. The change in new drug research and development provides an opportunity to develop TCM. TCM is based on a sophisticated system of medical theory and most traditional therapeutic formulae consist of a combination of several drugs. They are great treasures for the development of combination drugs. Unfortunately, there is little evidence (including clinical data) that provide a strong scientific basis to justify mixing plant extracts to improve pharmacological efficacy in clinical treatment. But it must be noted that they do indeed exist [64], [65]. The detailed action mechanisms of combination drugs from TCM stand out a major challenge. Moreover, the selection of the optimal combination and doses of ingredients in formulae remains a matter of trial and error [66]. One of the major challenges of combination therapy and drug discovery is the lack of effective evaluation methods. Since the 1950s, data mining methods from mathematics, statistics, and other computational sciences have been gradually introduced into TCM studies, making this modality more scientific in nature. Meanwhile, the distinct features of TCM theories and diagnostic model have constantly promoted the development of statistical methodologies [67].

Correct target identification and subsequent pharmacological manipulation of targets may give great help in the prevention and/or treatment of a number of the most prevalent diseases, including neurodegenerative disorders [68]. Strategies to antagonize injurious biochemical and molecular events that eventuate in irreversible injury in ischemic stroke are required [69]. Recanalization and neuroprotection are the two major approaches used to treat ischemic stroke. Considering the limitations related to its narrow therapeutic time window and concerns of hemorrhagic conversion, thrombolysis with a tissue plasminogen activator (tPA) is limited [8]. It is inevitable that thrombolysis must be evaluated clinically in combination with neuroprotectant agents. Based on the symptoms and characteristics of ischemic stroke patients and guided by TCM theories, formulae are designed to contain a combination of different types of plants or minerals to improve the clinical efficacy of treatment. It may be possible to find a combination of vasoactive drugs and neuroprotective agents in TCM formulae.

In the last decade, mitochondria have provided a vast area of research in pharmacology; a wealth of potential targets for drug action had been identified. A large body of research has demonstrated that MCAO induces marked mitochondrial dysfunction, including mitochondrial transition pore opening, membrane potential depolarization, and so on. Protection of mitochondrial function is closely related to stroke treatment [70]–[72]. In this study, we made use of mitochondrial function to evaluate the integrated effect and related mechanism of YJ on the treatment of acute ischemic stroke.

There may be an optimal proportion of formulaic ingredients to produce the best pharmacological action. To seek it, a new multi-objective regression algorithm was proposed based on LARS-PLS to construct the predictive model of pharmacological activity of YJ. Additionally, the optimal proportion of three compounds was found to be 3 (G): 2 (B): 0.5 (J), which was determined by PSO based on the predictive model mentioned above. Due to the limit of pharmacological experiments, it is hard to perform more than ten groups simultaneously, which means that the size of samples for further analysis is extremely small. When mathematical models were applied to it, it is easy to over fit the small data. Furthermore, a pharmacological index is computed as mean value of several (usually more than six) animals. Generally speaking, for small size data, the fitting accuracy is nearly 100%, but the predictive performance is sometimes notorious. In order to avoid this, we used two strategies. The one is to use LARS associated algorithm to deal with this small size data. The other is to sacrifice some fitting accuracy in order to compensate some predictive accuracy. The successful application of these two strategies provided a strong basis for multi-objection optimization here [73], [74].

To verify the precision of our predictive model, we conducted a confirmatory experiment of the effect of YJ. In this experiment, YJ obviously favored the improvement of mitochondrial structure and function, as indicated by the attenuation of mitochondrial swelling and membrane viscosity, amelioration of the reduced mitochondrial membrane potential state, and inhibition of cerebral ischemia-induced mitochondrial dysfunction at 24 h after MCAO.

To clarify whether YJ in the optimal 3∶2∶0.5 proportion could exhibit significant pharmacological effects on acute ischemic stroke, we further determined the accuracy of the predictive model. We performed the pharmacological experiments both in animal models and the molecular level. It is common sense that cerebral ischemia can cause brain injury that may leading to neurology defects, cerebral infarction and neuronal death by apoptosis and/or necrosis [75]. Our results indicated that YJ at doses of 1, 5 and 25 mg•kg^−1^ exhibited significant anti-cerebral ischemia activity at 12 h and 24 h after focal cerebral ischemia in a MCAO rat model, both at PM and AM. We demonstrated that YJ reduces infarct volumes after cerebral ischemic injury. Protection appeared in the early stage of acute ischemic stroke, and it is associated with improvement of the neurological deficits and cerebral edema resulting from arterial occlusion at PM and AM. Cerebral blood flow (CBF) derangements play key roles in the development of brain damage following cerebral ischemia [76]. Therefore, improved CBF has been proposed as one of the main strategies to limit ischemic injury [77]. Intriguingly, after YJ was administered, there was a remarkable improvement in rCBF after MCAO, providing strong support for its use in acute ischemic stroke.

Mitochondria play a key role in many apoptotic cascades and apoptotic cell death [78]. The mitochondrial pathway is closely associated with cell injury in cerebral ischemia. Following mitochondrial protection, YJ sequentially inhibits caspase-dependent (cytochrome c and caspase-3) mitochondrial cell death pathways. In the caspase-dependent manner, the release of cytochrome c activates the caspase cascade [4]. YJ at doses of 1, 5 and 25 mg•kg^−1^ inhibited the activation of caspase-3, decreased Bax expression, increased Bcl-2 expression and maintained the balance of pro- and anti-apoptotic proteins at 24 h after MCAO. Moreover, the markedly reduced release of cytochrome c from mitochondria as a result of YJ after the MCAO was also detected. Furthermore, ultrastructure observation results showed that YJ could improve the injuried mitochondrial and neuronic morphologies at the dose of 25 mg•kg^−1^.

So far, we proposed the three components and the optimal proportion of these components in YJ as a combination drug for the first time with the assistance of mathematical methods. Given that the YJ performed so well in terms of pharmacodynamics, we introduced the PLS VIP and synergistic experimental designs to explore the potential combination mechanism. The results of the PLS VIP suggested that J was the most important component in the formula. The principal components analysis results showed that the G, B and J combination could exhibit the strongest integrated pharmacological effect among all other possible combinations, suggesting that the three components are all indispensable. Among the mono- or bi-therapy combinations of G, B and J, B combined with J performed best. B, an isoquinoline alkaloid extracted from medicinal herbs, has been historically used as an antipyretic, antidiarrheal, bactericide and anti-inflammatory agent [79]. Recently, it has been reported that B has multiple neuropharmacological properties, such as anti-neuronal apoptosis effects [80]. The effect of J is manifested as detoxification, which can repair damage to vascular endothelial cells and block the progression of cascading damage observed in cerebral ischemia [81]. G is a tonic, which could improve the overall state of the organism. Abundant literature has revealed that G could alleviate many central nervous system disorders, including ischemic stroke [82]. Though J was only in small amounts in YJ, our data indicated that J was the key component of this formula. J counteracted the cardinal pathological effects of acute ischemic stroke. All of these results indicated that in the treatment of acute ischemic stroke, we should pay more attention to the removal of the toxic metabolites.

EGb761 is an extract of the leaf of Ginkgo biloba L. that is neuroprotective against focal cerebral ischemic injury [83], [84]. It has also been reported to protect against mitochondrial dysfunction [85] and inhibit the mitochondria-dependent caspase-apoptosis pathway [86]. Therefore, EGb761 was selected as the positive control for this study.

## Conclusions

In conclusion, with the help of mathematical methods and correctly identified targets, this study provided comprehensive evidence supporting the combination drug YJ for the treatment of acute ischemic stroke. Positive pharmacological effects of YJ suggested that PSO based on the LARS-PLS predictive model was relatively precise in exploring the optimal proportion of YJ ingredients. The pharmacodynamics of YJ on MCAO showed that YJ was a combination therapy drug, which was related to improvement of vascular environment and neuroprotective mechanisms. YJ played a direct neuroprotection role against ischemic injury, and its therapeutic effect could be expressed via mitochondrial protection and inhibition of the caspase-dependent mitochondrial cell death pathway. Furthermore, YJ could restore regional cortical blood perfusion after MCAO. Moreover, the analysis of PLS VIP and synergistic effects suggested that in the acute stage of ischemic stroke, except for improvement of vascular and nerves environment, the removal of the toxic metabolites is essential for treatment. Our data may provide theoretical support for TCM in the clinical treatment of acute ischemic stroke.

For the research and development of combination drugs for complex diseases, powerful mathematical and statistical methods may be indispensable. Furthermore, the therapeutic formulae from TCM are great treasures for the development of combination drugs. It is expected that this study will provide new insights to the development of combination drugs using TCM. This work may provide a viable novel mode for the research and development of new combination drugs.

## Supporting Information

Figure S1 **The chromatogram of Rb_2_.**
(TIF)

Figure S2 **The chromatogram of Rd.**
(TIF)

Figure S3 **The chromatogram of Re.**
(TIF)

Figure S4 **The chromatogram of Rg_1_.**
(TIF)

Figure S5 **The chromatogram of test sample solution (20110510).**
(TIF)

Figure S6 **The chromatogram of test sample solution (20110530).**
(TIF)

Figure S7 **The chromatogram of test sample solution (20110612).**
(TIF)

Figure S8 **The chromatogram of Rb_1_.**
(TIF)

Figure S9 **The chromatogram of Rb_3_.**
(TIF)

Figure S10 **The chromatogram of Rc.**
(TIF)

Figure S11 **The chromatogram of F_1_.**
(TIF)

Figure S12 **The chromatogram of F_2_.**
(TIF)

Figure S13 **The chromatogram of Rg_3_.**
(TIF)

Figure S14 **The chromatogram of test sample solution (20110510).**
(TIF)

Figure S15 **The chromatogram of test sample solution (20110530).**
(TIF)

Figure S16 **The chromatogram of test sample solution (20110612).**
(TIF)

Figure S17 **The mass spectrum of the Rg_1_ solution and the test sample solution.** a. Rg_1_; b. the batch of 20110510; c. the batch of 20110530; d. the batch of 20110612.
(TIF)

Figure S18 **The mass spectrum of the Re solution and the test sample solution.** a. Re; b. the batch of 20110510; c. the batch of 20110530; d. the batch of 20110612.
(TIF)

Figure S19 **The mass spectrum of the Rd solution and the test sample solution.** a. Rd; b. the batch of 20110510; c. the batch of 20110530; d. the batch of 20110612.
(TIF)

Figure S20 **The mass spectrum of the Rb_1_ solution and the test sample solution.** a. Rb_1_; b. the batch of 20110510; c. the batch of 20110530; d. the batch of 20110612.
(TIF)

Figure S21 **The mass spectrum of the Rb_2_ solution and the test sample solution.** a. Rb_2_; b. the batch of 20110510; c. the batch of 20110530; d. the batch of 20110612.
(TIF)

Figure S22 **The mass spectrum of the Rb_3_ solution and the test sample solution.** a. Rb_3_; b. the batch of 20110510; c. the batch of 20110530; d. the batch of 20110612.
(TIF)

Figure S23 **The mass spectrum of the F_1_ solution and the test sample solution.** a. F_1_; b. the batch of 20110510; c. the batch of 20110530; d. the batch of 20110612.
(TIF)

Figure S24 **The mass spectrum of the F_2_ solution and the test sample solution.** a. F_2_; b. the batch of 20110510; c. the batch of 20110530; d. the batch of 20110612.
(TIF)

Figure S25 **The mass spectrum of the Rc solution and the test sample solution.** a. Rc; b. the batch of 20110510; c. the batch of 20110530; d. the batch of 20110612.
(TIF)

Figure S26 **The mass spectrum of the Rg_3_ solution and the test sample solution.** a. Rg_3_; b. the batch of 20110510; c. the batch of 20110530; d. the batch of 20110612.
(TIF)

Text S1 **Supporting Information Legends.**
(DOC)

Table S1 **THILE Index and RMSE for BPNN regression model.**
(TIF)

Table S2 **Comparison of THILE Index and RMSE among the four models.**
(TIF)

Table S3 **The peak area of each ginsenosides standards.**
(TIF)

Table S4 **The peak area of each ginsenosides standards in three batches.**
(TIF)

Table S5 **The content of each ginsenosides standards in three batches.**
(TIF)

## References

[1] Xutian S, Zhang J, Louise W (2009) New exploration and understanding of traditional Chinese medicine. Am. J. Chin. Med. 37: 411–426. 10.1142/S0192415X09006941 19606504

[2] Li S, Zhang B, Zhang N (2011) Network target for screening synergistic drug combinations with application to traditional Chinese medicine. BMC. Syst. Biol. 5 Suppl 1S10. 10.1186/1752-0509-5-S1-S10 PMC3121110 21689469

[3] Wang L, Zhou GB, Liu P, Song JH, Liang Y, et al (2008) Dissection of mechanisms of Chinese medicinal formula Realgar-Indigo naturalis as an effective treatment for promyelocytic leukemia. Proc. Natl. Acad. Sci. USA. 105: 4826–4831. 10.1073/pnas.0712365105 PMC2290784 18344322

[4] Vosler PS, Graham SH, Wechsler LR, Chen J (2009) Mitochondrial targets for stroke: focusing basic science research toward development of clinically translatable therapeutics. Stroke. 40: 3149–3155. 10.1161/STROKEAHA.108.543769 PMC2733936 19478227

[5] Kannan K, Holcombe RF, Jain SK, Alvarez-Hernandez X, Chervenak R, et al (2000) Evidence for the induction of apoptosis by endosulfan in a human T-cell leukemic line. Mol. Cell. Biochem. 205: 53–66. 10.1023/a:1007080910396 10821422

[6] Blomgren K, Zhu C, Hallin U, Hagberg H (2003) Mitochondria and ischemic reperfusion damage in the adult and in the developing brain. Biochem. Biophys. Res. Commun. 304: 551–559. 10.1016/s0006-291x(03)00628-4 12729590

[7] Sims NR, Anderson MF (2002) Mitochondrial contributions to tissue damage in stroke. Neurochem. Int. 40: 511–526. 10.1016/s0197-0186(01)00122-x 11850108

[8] Ye R, Zhang X, Kong X, Han J, Yang Q, et al (2011) Ginsenoside Rd attenuates mitochondrial dysfuction and sequential apoptosis after transient focal ischemia. Neuroscience. 178: 169–180. 10.1016/j.neuroscience.2011.01.007 21219973

[9] Hata R, Mies G, Wiessner C, Fritze K, Hesselbarth D, et al (1998) A reproducible model of middle cerebral artery occlusion in mice: hemodynamic, biochemical, and magnetic resonance imaging. J. Cereb. Blood. Flow. Metab. 18: 367–375. 10.1097/00004647-199804000-00004 9538901

[10] Lindvall O, Kokaia Z (2004) Recovery and rehabilitation in stroke: stem cells. Stroke. 35: 2691–2694. 10.1161/01.STR.0000143323.84008.f4 15459434

[11] Zhang ZJ, Li P, Wang Z, Li PT, Zhang WS, et al (2006) A comparative study on the individual and combined effects of baicalin and jasminoidin on focal cerebral ischemia-reperfusion injury. Brain Res. 1123: 188–195. 10.1016/j.brainres.2006.09.063 17069775

[12] Yang L, Xu S, Liu C, Su Z (2009) In vivo metabolism study of ginsenoside Re in rat using high-performance liquid chromatography coupled with tandem mass spectrometry. Anal Bioanal Chem. 395: 1441–1451. 10.1007/s00216-009-3121-1 19774367

[13] Nakaya Y, Mawatari K, Takahashi A, Harada N, Hata A, et al (2007) The phytoestrogen ginsensoside Re activates potassium channels of vascular smooth muscle cells through PI3K/Akt and nitric oxide pathways. J Med Invest. 54: 381–384. 10.2152/jmi.54.381 17878692

[14] Radad K, Moldzio R, Rausch WD (2011) Ginsenosides and their CNS targets. CNS Neurosci Ther. 17: 761–768. 10.1111/j.1755-5949.2010.00208.x PMC6493809 21143430

[15] Zheng GQ, Cheng W, Wang Y, Wang XM, Zhao SZ, et al (2011) Ginseng total saponins enhance neurogenesis after focal cerebral ischemia. J Ethnopharmacol. 133: 724–728. 10.1016/j.jep.2010.01.064 21073942

[16] Leung KW, Leung FP, Huang Y, Mak NK, Wong RN (2007) Nongenomic effects of ginsenoside-Re in endothelial cells via glucocorticoid receptor. FEBS Lett. 581: 2423–2428. 10.1016/j.febslet.2007.04.055 17490654

[17] Zhou XM, Cao YL, Dou DQ (2006) Protective effect of ginsenoside-Re against cerebral ischemia/reperfusion damage in rats. Biol Pharm Bull. 29: 2502–2505. 10.1248/bpb.29.2502 17142990

[18] Kettmann V, Kosfalova D, Jantova S, Cernakova M, Drimal J (2004) In vitro cytotoxicity of berberine against Hela and L1210 cancer cell lines. Pharmazie. 59: 548–551. 15296093

[19] Tran QL, Tezuka Y, Ueda JY, Nquyen NT, Maruyama Y, et al (2003) In vitro antiplasmodial activity of antimalatial medicinal plants used in Vietnamese traditional medicine. J Ethnopharmacol. 86: 249–252. 10.1016/s0378-8741(03)00045-x 12738095

[20] Zhou XQ, Zeng XN, Kong H, Sun XL (2008) Neuroprotective effects of berberine on stroke models in vitro and in vivo. Neurosci Lett. 447: 31–36. 10.1016/j.neulet.2008.09.064 18838103

[21] Suzuki Y, Kondo K, Ikeda Y, Umemura K (2001) Antithrombotic effect of geniposide and genipin in the mouse thrombosis model. Planta Med. 67: 807–810. 10.1055/s-2001-18842 11745015

[22] Koo HJ, Lee S, Shin KH, Kim BC, Lim CJ, et al (2004) Geniposide, anti-angiogenic compound from the fruits of Gardenia jasminoitrdes. Planta Med. 70: 467–469. 10.1055/s-2004-818978 15124095

[23] Kuo WH, Wang CJ, Young SC, Sun YC, Chen YJ, et al (2004) Differential induction of the expression of GST subunits by geniposide in rat hepatocytes. Pharmacology. 70: 15–22. 10.1159/000074238 14646352

[24] Zhu XL, Zhang N, Li PT, Jiang YF, Xu Y (2004) Protective effect of jasminoidin on cascade of damage of cerebral ischemia in rats. Zhongguo Zhong Yao Za Zhi. 29: 1065–1068. 15656140

[25] Khanal T, Kim HG, Choi JH, Do MT, Kong MJ, et al (2012) Biotransformation of geniposide by human intestinal microflora on cytotoxicity against HepG2 cells. Toxicol Lett. 109: 246–254. 10.1016/j.toxlet.2011.12.017 22245672

[26] Liu J, Yin F, Xiao H, Guo L, Gao X (2012) Glucagon-like peptide 1 receptor plays an essential role in geniposide attenuating lipotoxicity-induced β-cell apoptosis. Toxicol In Vitro. 26: 1093–1097. 10.1016/j.tiv.2012.07.004 22819839

[27] Kuo WH, Wang CJ, Young SC, Sun YC, Chen YJ, et al (2004) Differential induction of the expression of GST subunits by geniposide in rat hepatocytes. Pharmacology. 70: 15–22. 10.1159/000074238 14646352

[28] Datta SR, Brunet A, Greenberg ME (1999) Cellular survival: a play in three Akts. Genes Dev 13: 2905–2927. 10579998 10.1101/gad.13.22.2905

[29] Zhang X, Zhang X, Wang C, Li Y, Dong L, et al (2012) Neuroprotection of early and short-time applying berberine in the acute phase of cerebral ischemia: up-regulated pAkt, pGSK and pCREB, down-regulated NF-κB expression, ameliorated BBB permeability. Brain Res. 1459: 61–70. 10.1016/j.brainres.2012.03.065 22560097

[30] Hu J, Chai Y, Wang Y, Kheir MM, Li H, et al (2012) PI3K p55γ promoter activity enhancement is involved in the anti-apoptotic effect of berberine against cerebral ischemia-reperfusion. Eur J Pharmacol. 674: 132–142. 10.1016/j.ejphar.2011.11.014 22119079

[31] Dancey JE, Chen HX (2006) Strategies for optimizing combinations of molecularly targeted anticancer agents. Nat Rev Drug Discov. 5: 649–659. 10.1038/nrd2089 16883303

[32] Jiang WL, Zhang SP, Zhu HB, Hou J, Tian JW (2010) Cornin ameliorates cerebral infarction in rats by antioxidant action and stabilization of mitochondrial function. Phytother Res. 24: 547–552. 10.1002/ptr.2978 20041427

[33] Wang DG (2009) Numerical analysis of the validity of uniform design in stated choice modeling. Transport Reviews. 29: 619–634.

[34] Fang KT, Dennis KJLin (2003) Uniform experimental designs and their applications in industry. Handbook of statistics. 22: 131–170.

[35] Xia T, Jiang C, Li L, Wu C, Chen Q, et al (2002) A study on permeability transition pore opening and cytochrome c release from mitochondria, induced by caspase-3 in vitro. FEBS Lett. 510: 62–66. 10.1016/s0014-5793(01)03228-8 11755532

[36] Zhang HX, Du GH, Zhang JT (2004) Assay of mitochondrial functions by resazurin in vitro. Acta Pharmacol Sin. 25: 385–389. 15000895

[37] Tian J, Fu F, Geng M, Jiang Y, Yang J, et al (2005) Neuroprotective effect of 20(S)-ginsenoside Rg3 on cerebral ischemia in rats. Neurosci Lett. 374: 92–97. 10.1016/j.neulet.2004.10.030 15644271

[38] Chen LM, Zhou XM, Cao YL, Hu WX (2008) Neuroprotection of ginsenoside Re in cerebral ischemia-reperfusion injury in rats. J Asian Nat Prod Res. 10: 439–445. 10.1080/10286020801892292 18464084

[39] Hirano K (1991) Change in membrane uidity of sand dollar egg cortices caused by Ca-induced exocytosis: microscopic analysis with uorescence anisotropy. Dev Growth Differ. 33: 451–458. 10.1111/j.1440-169X.1991.00451.x 37282321

[40] Zhang HX, Du GH, Zhang JT (2003) Ischemic pre-conditioning preserves brain mitochondrial functions during the middle cerebral artery occlusion in rat. Neurol Res. 25: 471–476. 10.1179/016164103101201878 12866194

[41] He XL, Wang YH, Gao M, Li XX, Zhang TT, et al (2009) Baicalein protects rat brain mitochondria against chronic cerebral hypoperfusion-induced oxidative damage. Brain Res. 1249: 212–221. 10.1016/j.brainres.2008.10.005 18977207

[42] Ma H, Quan F, Chen D, Zhang B, Zhang Y (2010) Alterations in mitochondrial function and spermatozoal motility in goat spermatozoa following incubation with a human lysozyme plasmid. Anim Reprod Sci. 121: 106–114. 10.1016/j.anireprosci.2010.05.005 20638954

[43] Gilan SS, Jovein HB, Ramezanianpour AA (2012) Hybrid support vector regression – Particle swarm optimization for prediction of compressive strength and RCPT of concretes containing metakaolin. Constr Build Mater. 34: 321–329.

[44] Peng J, Peng Y, Ouyang LN (2008) Hybrid particle swarm optimization and support vector machine for bankruptcy prediction. Journal of Shanghai Jiaotong University. 42: 189–194.

[45] Appice A, Dzeroski S (2007) Stepwise induction of multi-target model trees. Lecture Notes in Computer Science. 4701: 502–509.

[46] Adebiyi OA, Corripio AB (2003) Dynamic neural networks partial least squares (DNNPLS) identification of multivariable processes. Computers and Chemical Engineering. 27: 143–155.

[47] Zou H (2006) The adaptive lasso and its oracle properties. Journal of the American Statistical Association. 101: 1418–1429.

[48] Efron B, Hastie T, Johnstone I, Tibshirani R (2004) Least angle regression. The Annals of Statistics. 32: 407–451.

[49] Geladi P, Kowalski BR (1986) Partialleast-squares regression: A Tutorial. Analytica Chimica Acta. 185: 1–17.

[50] Malthouse EC, Tamhane AC, Mah RSH (1997) Nonlinear partial least squares. Computers and Chemical Engineering. 21: 875–890.

[51] Qin SJ, Mc Avoy TJ (1992) Nonlinear PLS modeling using neural networks. Computers and Chemical Engineering. 16: 379–391.

[52] Kennedy J, Eberhart RC (1995) Particle swarm optimization, in Proc. IEEE Int. Conf. Neural Networks. 1942–1948.

[53] Eberhart RC, Shi YH, Kennedy J (2001) Swarm intelligence. Morgan Kaufman.

[54] Longa EZ, Weinstein PR, Carlson S, Cummins R (1989) Reversible middle cerebral artery occlusion without craniectomy in rats. Stroke. 20: 84–91. 10.1161/01.str.20.1.84 2643202

[55] Saraf MK, Prabhakar S, Anand A (2010) Neuroprotective effect of Bacopa monniera on ischemia induced brain injury. Pharmacol Biochem Behav. 97: 192–197. 10.1016/j.pbb.2010.07.017 20678517

[56] Shi LL, Chen BN, Gao M, Zhang HA, Li YJ, et al (2011) The characteristics of therapeutic effect of pinocembrin in transient global brain ischemia/reperfusion rats. Life Sci. 88: 521–528. 10.1016/j.lfs.2011.01.011 21262238

[57] Boas DA, Dunn AK (2010) Laser speckle contrast imaging in biomedical optics. J Biomed Opt. 15: 011109. 10.1117/1.3285504 PMC2816990 20210435

[58] Briers JD (2001) Laser Doppler, speckle and related techniques for blood perfusion mapping and imaging. Physiol Meas. 22: R35–66. 10.1088/0967-3334/22/4/201 11761081

[59] Draijer M, Hondebrink E, van Leeuwen T, Steenbergen W (2009) Review of laser speckle contrast techniques for visualizing tissue perfusion. Lasers Med Sci. 24: 639–651. 10.1007/s10103-008-0626-3 PMC2701498 19050826

[60] Gao M, Liu R, Zhu SY, Du GH (2008b) Acute neurovascular unit protective action of pinocembrin against permanent cerebral ischemia in rats. J Asian Nat Prod Res. 10: 551–558. 10.1080/10286020801966955 18470808

[61] Umetrics AB (2006) Multi- and Megavariate Data Analysis, part 1, Basic principles and applications. Vol.2. Sweden: MKS Umetrics AB.

[62] Tan CB, Gao M, Xu WR, Yang XY, Zhu XM, et al (2009) Protective effects of salidroside on endothelial cell apoptosis induced by cobalt chloride. Biol Pharm Bull. 32: 1359–1363. 10.1248/bpb.32.1359 19652374

[63] Sucher NJ (2006) Insights from molecular investigations of traditional Chinese herbal stroke medicines: Implications for neuroprotective epilepsy therapy. Epilepsy Behav. 8: 350–362. 10.1016/j.yebeh.2005.11.015 16455305

[64] Gertsch J (2011) Botanical drugs, synergy, and network pharmacology: forth and back to intelligent mixtures. Planta Med. 77: 1086–1098. 10.1055/s-0030-1270904 21412698

[65] Chen J, Ma X, Gao K, Wang Y, Zhao H, et al (2012) The active ingredients of Jiang-Zhi-Ning: study of the Nelumbo nucifera alkaloids and their main bioactive metabolites. Molecules. 17: 9855–9867. 10.3390/molecules17089855 PMC6268456 22898740

[66] Jonker DM, Visser SA, van der Graaf PH, Voskuyl RA, Danhof M (2005) Towards a mechanism-based analysis of pharmacodynamic drug-drug interactions in vivo. Pharmacol Ther. 106: 1–18. 10.1016/j.pharmthera.2004.10.014 15781119

[67] Hu J, Qiao J, Kang D, Liu B (2011) Analysis on the distinguishing features of traditional Chinese therapeutics and related statistical issues. Front Med. 5: 203–207. 10.1007/s11684-011-0138-6 21695626

[68] Chen J, Zhao H, Yang Y, Liu B, Ni J, et al (2011) Lipid-lowering and antioxidant activities of Jiang-Zhi-Ning in Traditional Chinese Medicine. J Ethnopharmacol. 134: 919–930. 10.1016/j.jep.2011.01.048 21316437

[69] Ginsberg MD (2008) Neuroprotection for ischemic stroke: past, present and future. Neuropharmacology. 55: 363–389. 10.1016/j.neuropharm.2007.12.007 PMC2631228 18308347

[70] Dave KR, DeFazio RA, Raval AP, Torraco A, Saul I, et al (2008) Ischemic preconditioning targets the respiration of synaptic mitochondria via protein kinase C epsilon. J Neurosci. 28: 4172–4182. 10.1523/JNEUROSCI.5471-07.2008 PMC2678917 18417696

[71] Zheng CY, Zhang HY, Tang XC (2008) Huperzine A attenuates mitochondrial dysfunction after middle cerebral artery occlusion in rats. J Neurosci Res. 86: 2432–2440. 10.1002/jnr.21681 18438924

[72] Shen H, Kuo CC, Chou J, Delvolve A, Jackson SN, et al (2009) Astaxanthin reduces ischemic brain injury in adult rats. FASEB J. 23: 1958–1968. 10.1096/fj.08-123281 PMC2698661 19218497

[73] Yang HJ, Chen JX, Tang SH, Li ZK, Zhen YS, et al (2009) New drug R&D of traditional Chinese medicine: Role of data mining approaches. Journal of Biological Systems. 17: 329–347.

[74] Chen C, Chen JX, Wu HW, Tang SH, Li G, et al (2011) Identification of key constituents in volatile oil of ligusticum chuanxiong based on data mining approaches. Pharmaceutical Biology 49: 445. 21501098 10.3109/13880209.2010.523426

[75] Iwanami J, Mogi M, Okamoto S, Gao XY, Li JM, et al (2007) Pretreatment with eplerenone reduces stroke volume in mouse middle cerebral artery occlusion model. Eur J Pharmacol. 566: 153–159. 10.1016/j.ejphar.2007.03.043 17475237

[76] Rudzinski W, Swiat M, Tomaszewski M, Krejza J (2007) Cerebral hemodynamics and investigations of cerebral blood flow regulation. Nucl Med Rev Cent East Eur. 10: 29–42. 17694500

[77] Della Morte D, Rave AP, Dave KR, Lin HW, Perez Pinzon MA (2011) Post-Ischemic Activation of protein kinase C epsilon protects the hippocampus from cerebral ischemic injury via alterations in cerebral blood flow. Neurosci Lett. 487: 158–162. 10.1016/j.neulet.2010.10.013 PMC3004991 20951185

[78] Everett H, Barry M, Sun X, Lee SF, Frantz C, et al (2002) The myxoma poxvirus protein, M11L, prevents apoptosis by direct interaction with the mitochondrial permeability transition pore. J Exp Med. 196: 1127–1139. 10.1084/jem.20011247 PMC2194110 12417624

[79] Benaissa F, Mohseni Rad H, Rahimi Moghaddam P, Mahmoudian M (2009) Berberine reduces the hypoxic-ischemic insult in rat pup brain. Acta Physiol Hung. 96: 213–220. 10.1556/APhysiol.96.2009.2.6 19457765

[80] Ye M, Fu S, Pi R, He F (2009) Neuropharmacological and pharmacokinetic properties of berberine: a review of recent research. J Pharm Pharmacol. 61: 831–837. 10.1211/jpp/61.07.0001 19589224

[81] Zhang G, He JL, Xie XY, Yu C (2012) LPS-induced iNOS expression in N9 microglial cells is suppressed by geniposide via ERK, p38 and nuclear factor-κB signaling pathways. Int J Mol Med. 30: 561–568. 10.3892/ijmm.2012.1030 22710392

[82] Lu T, Jiang Y, Zhou Z, Yue X, Wei N, et al (2011) Intranasal ginsenoside Rb1 targets the brain and ameliorates cerebral ischemia/reperfusion injury in rats. Biol Pharm Bull. 34: 1319–1324. 10.1248/bpb.34.1319 21804225

[83] Koh PO (2011) Gingko biloba extract (EGb 761) attenuates the focal cerebral ischemic injury-induced decrease in astrocytic phosphoprotein PEA-15 levels. Am. J. Chin. Med. 39: 971–979. 10.1142/S0192415X11009342 21905286

[84] Zhang Z, Peng D, Zhu H, Wang X (2012) Experimental evidence of Ginkgo biloba extract EGB as a neuroprotective agent in ischemia stroke rats. Brain. Res. Bull. 87: 193–198. 10.1016/j.brainresbull.2011.11.002 22100334

[85] Shi C, Xiao S, Liu J, Guo K, Wu F, et al (2010) Ginkgo biloba extract EGb761 protects against aging-associated mitochondrial dysfunction in platelets and hippocampi of SAMP8 mice. Platelets. 21: 373–379. 10.3109/09537100903511448 20459350

[86] Shen J, Lee W, Gu Y, Tong Y, Fung PC, et al (2011) Ginkgo biloba extract (EGb761) inhibits mitochondria-dependent caspase pathway and prevents apoptosis in hypoxia-reoxygenated cardiomyocytes. Chin. Med. 6: 8. 10.1186/1749-8546-6-8 PMC3053309 21345217

