# Supplementary figures and images for: An Effective Solution to Discover Synergistic Drugs for Anti-Cerebral Ischemia from Traditional Chinese Medicinal Formulae
Source: PLoS One. 2013 Nov 13;8(11):e78902. doi: 10.1371/journal.pone.0078902 (PMC3827340; doi:10.1371/journal.pone.0078902)

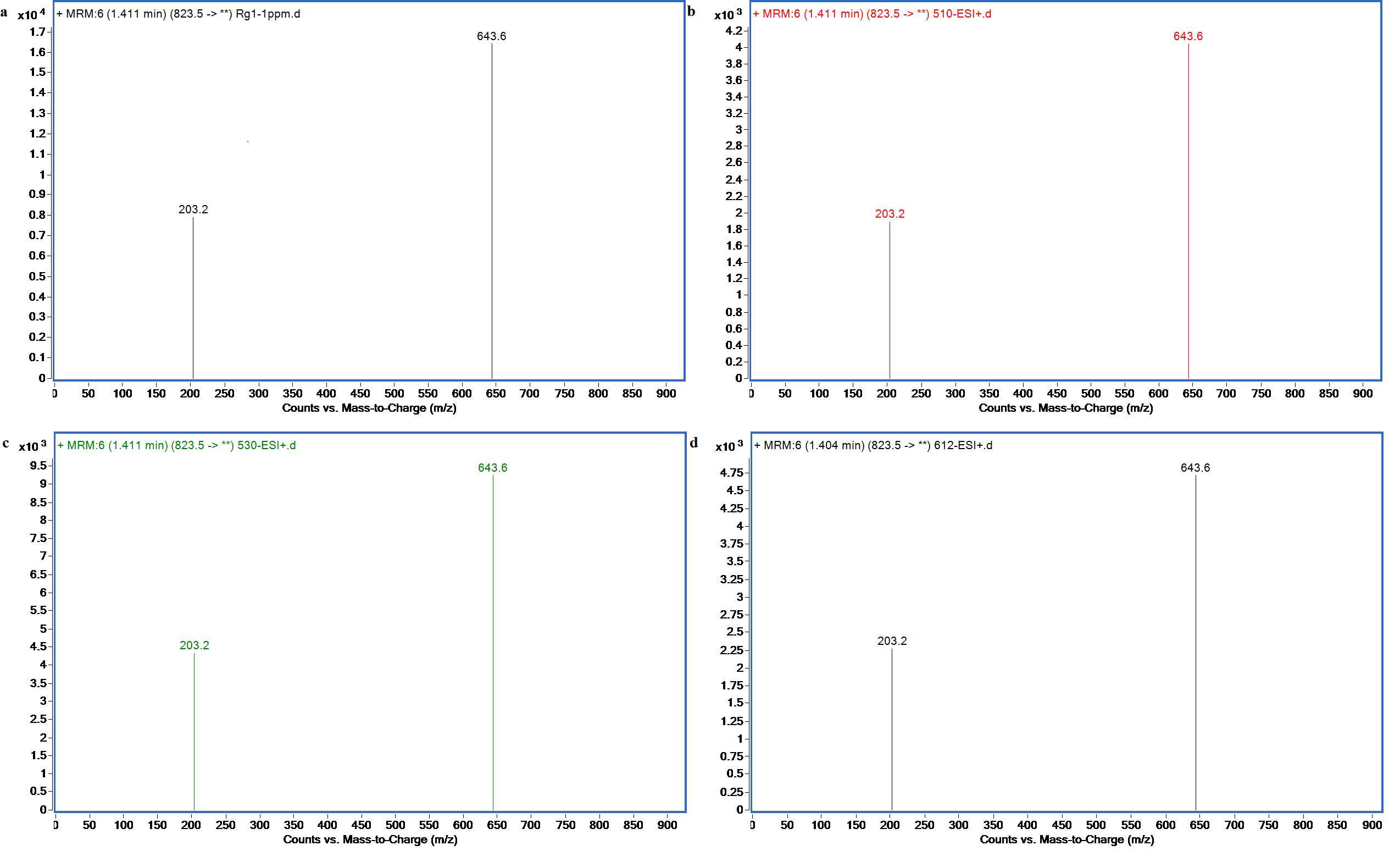

Supplement: Figure S17 — The mass spectrum of the Rg1 solution and the test sample solution. a. Rg1; b. the batch of 20110510; c. the batch of 20110530; d. the batch of 20110612. (TIF) [file pone.0078902.s017.tif]

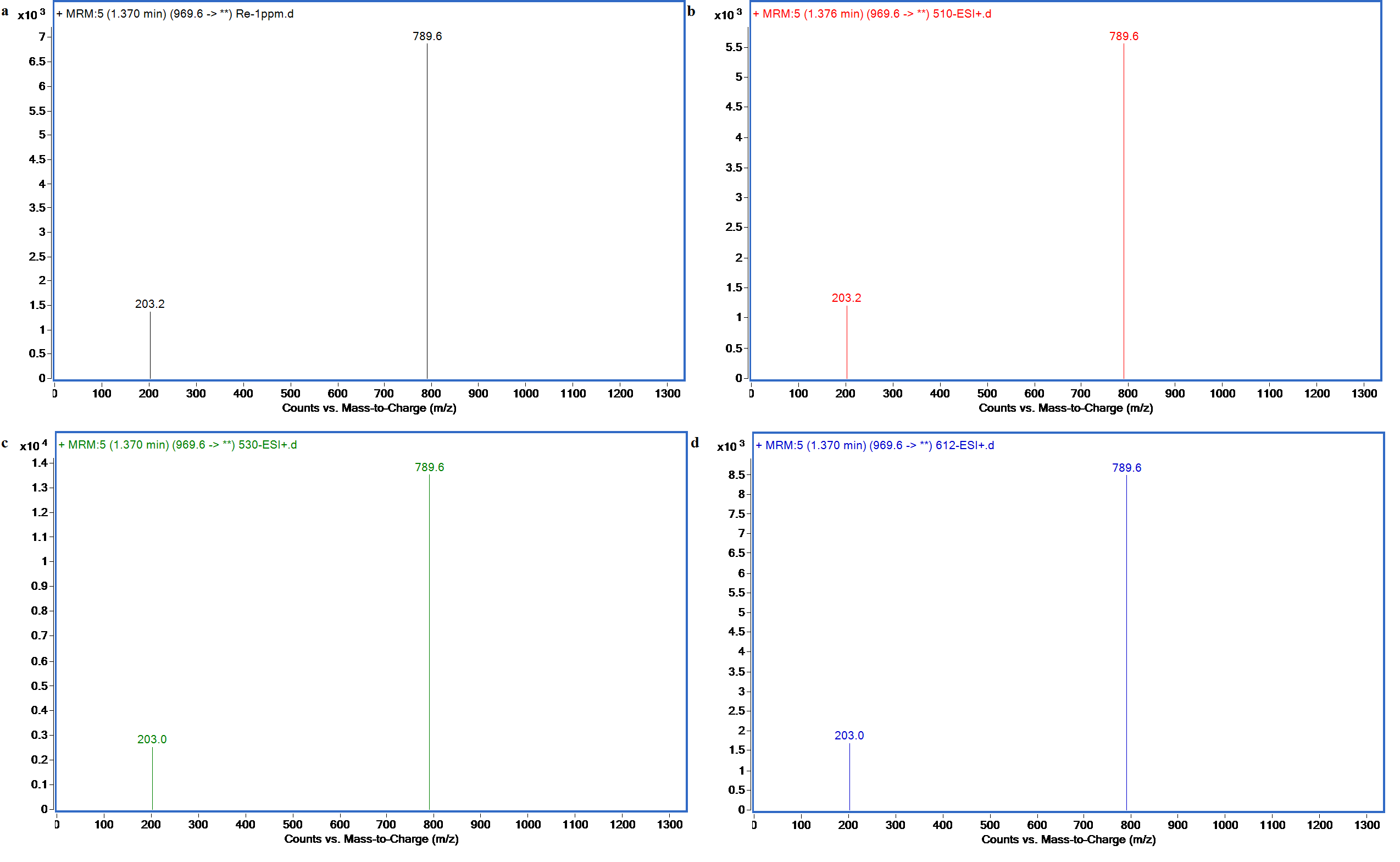

Supplement: Figure S18 — The mass spectrum of the Re solution and the test sample solution. a. Re; b. the batch of 20110510; c. the batch of 20110530; d. the batch of 20110612. (TIF) [file pone.0078902.s018.tif]

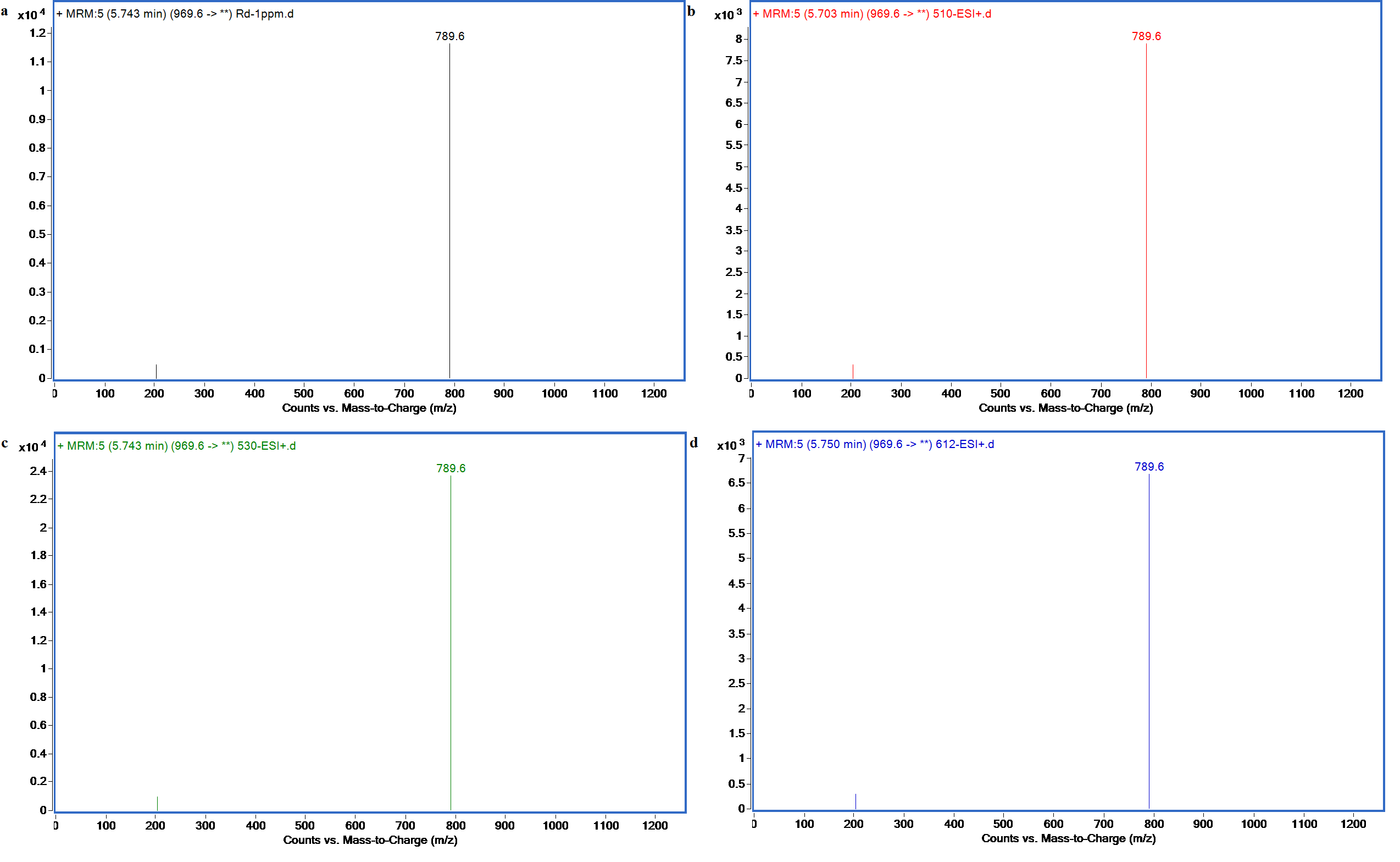

Supplement: Figure S19 — The mass spectrum of the Rd solution and the test sample solution. a. Rd; b. the batch of 20110510; c. the batch of 20110530; d. the batch of 20110612. (TIF) [file pone.0078902.s019.tif]

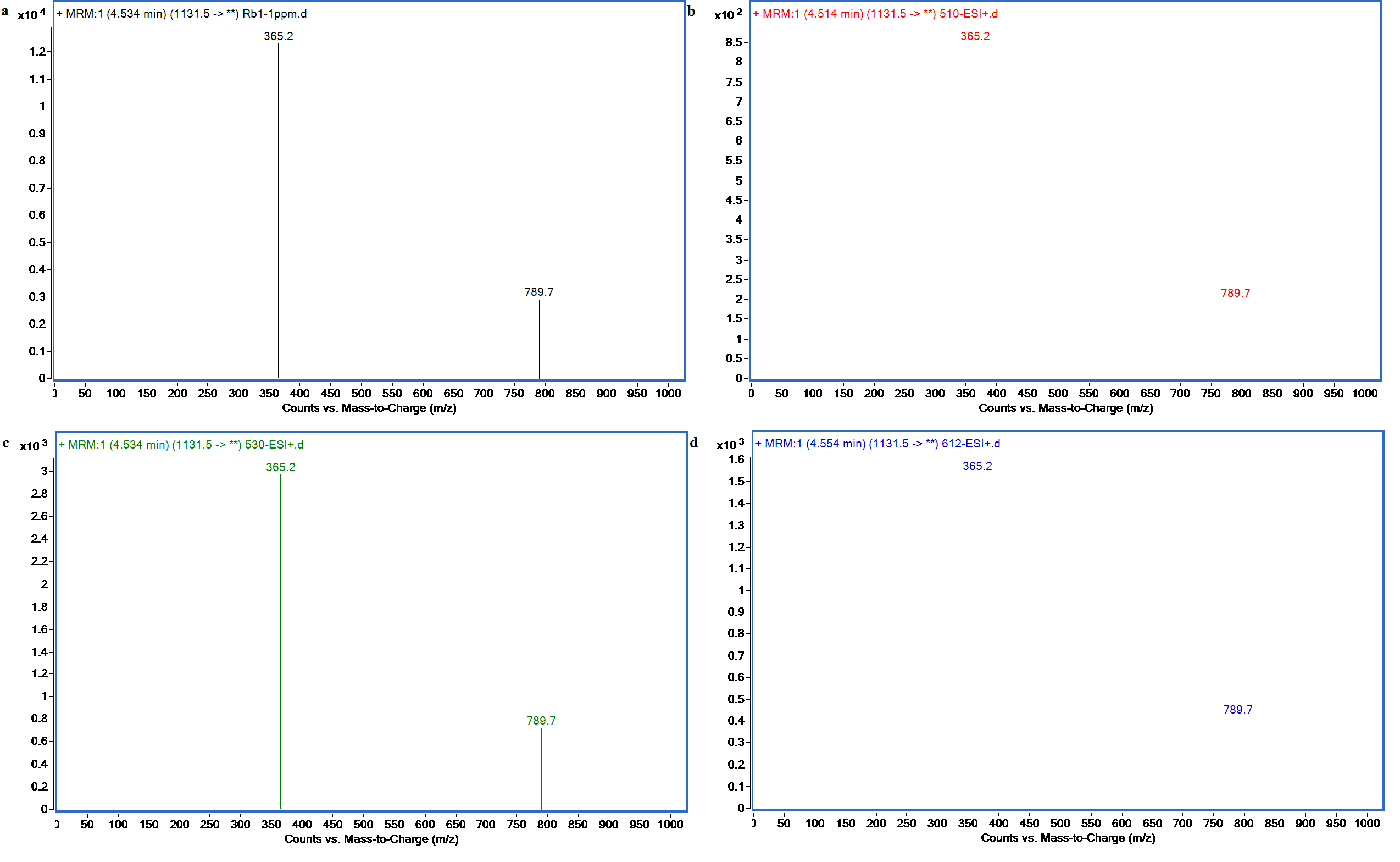

Supplement: Figure S20 — The mass spectrum of the Rb1 solution and the test sample solution. a. Rb1; b. the batch of 20110510; c. the batch of 20110530; d. the batch of 20110612. (TIF) [file pone.0078902.s020.tif]

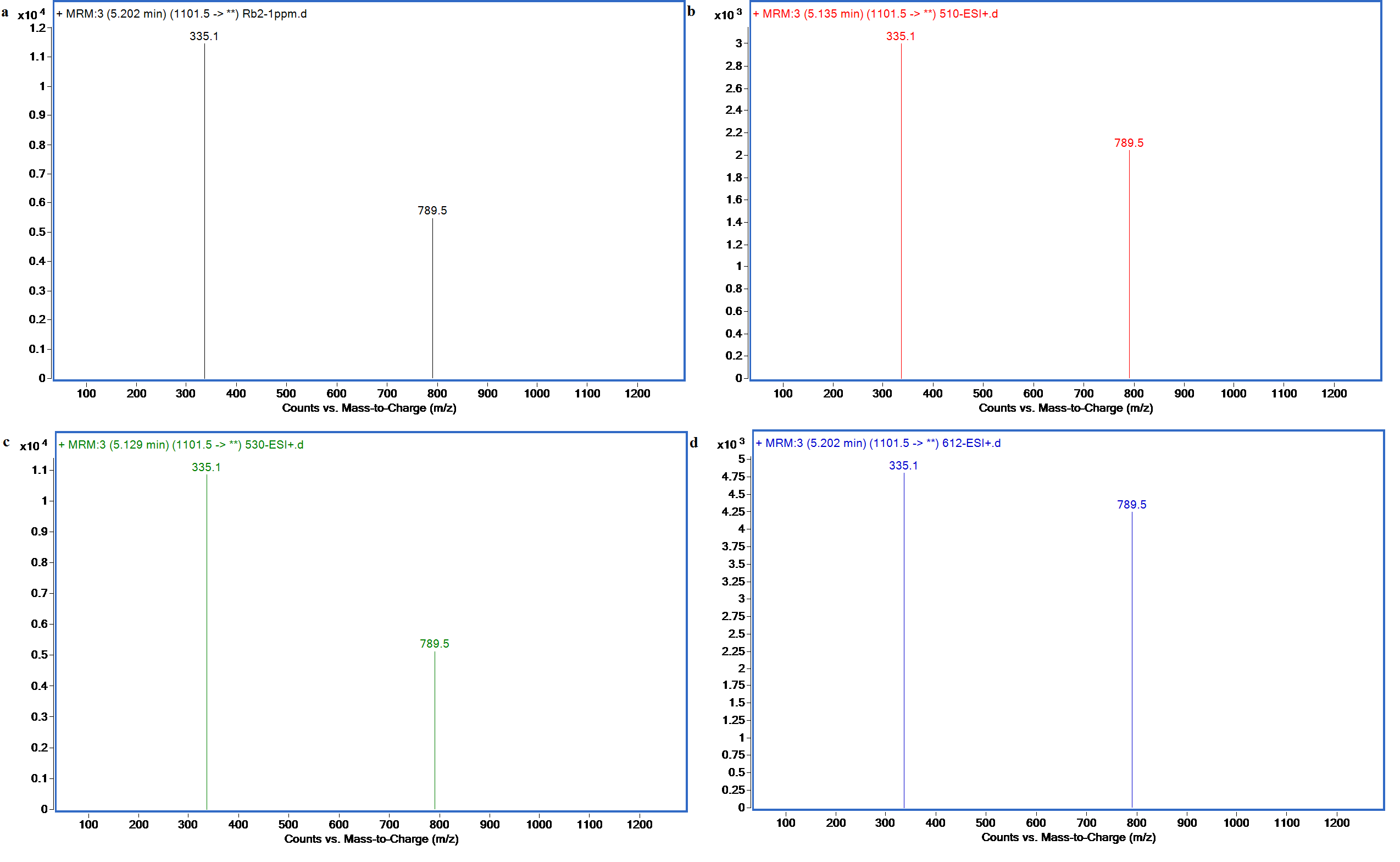

Supplement: Figure S21 — The mass spectrum of the Rb2 solution and the test sample solution. a. Rb2; b. the batch of 20110510; c. the batch of 20110530; d. the batch of 20110612. (TIF) [file pone.0078902.s021.tif]

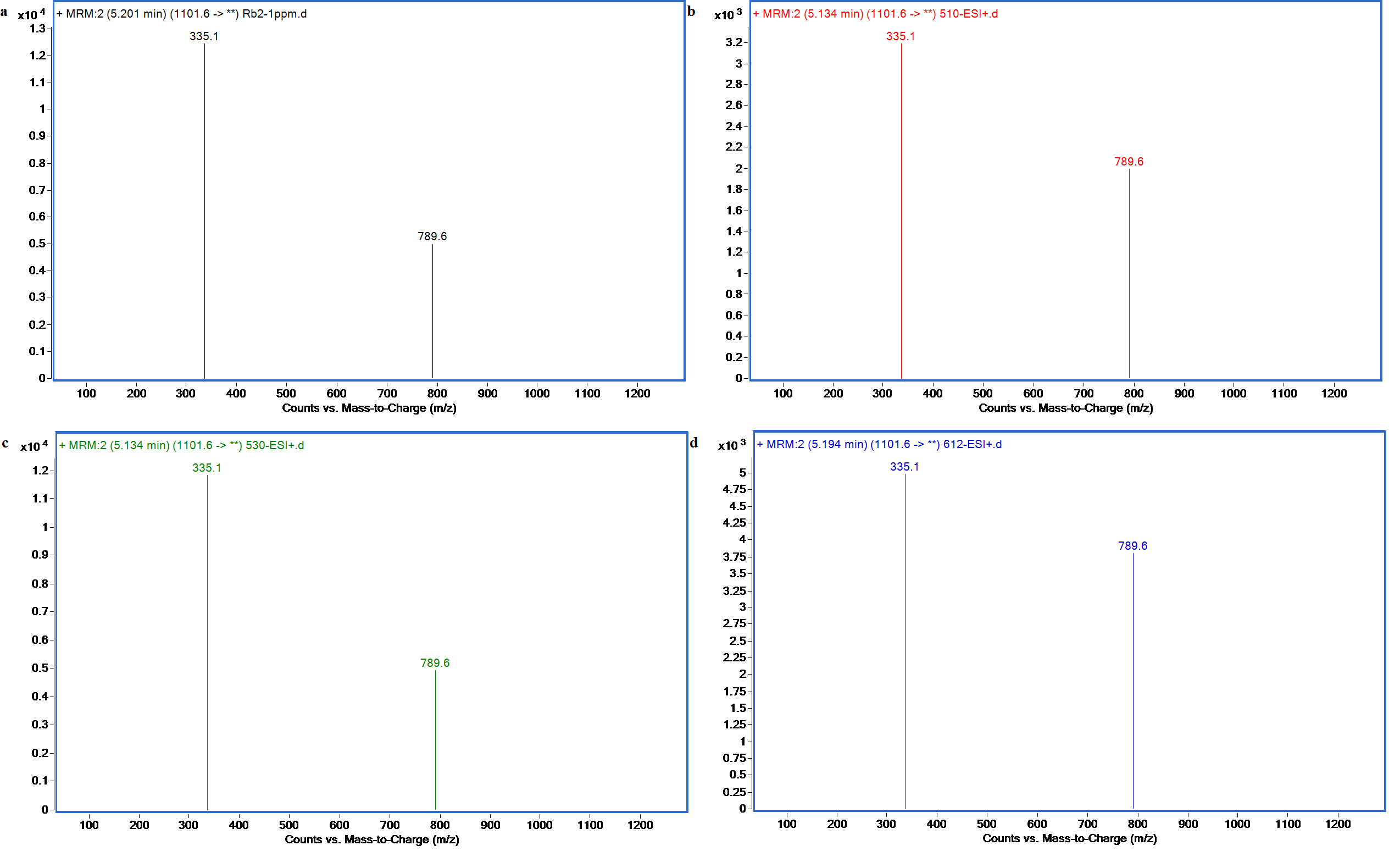

Supplement: Figure S22 — The mass spectrum of the Rb3 solution and the test sample solution. a. Rb3; b. the batch of 20110510; c. the batch of 20110530; d. the batch of 20110612. (TIF) [file pone.0078902.s022.tif]

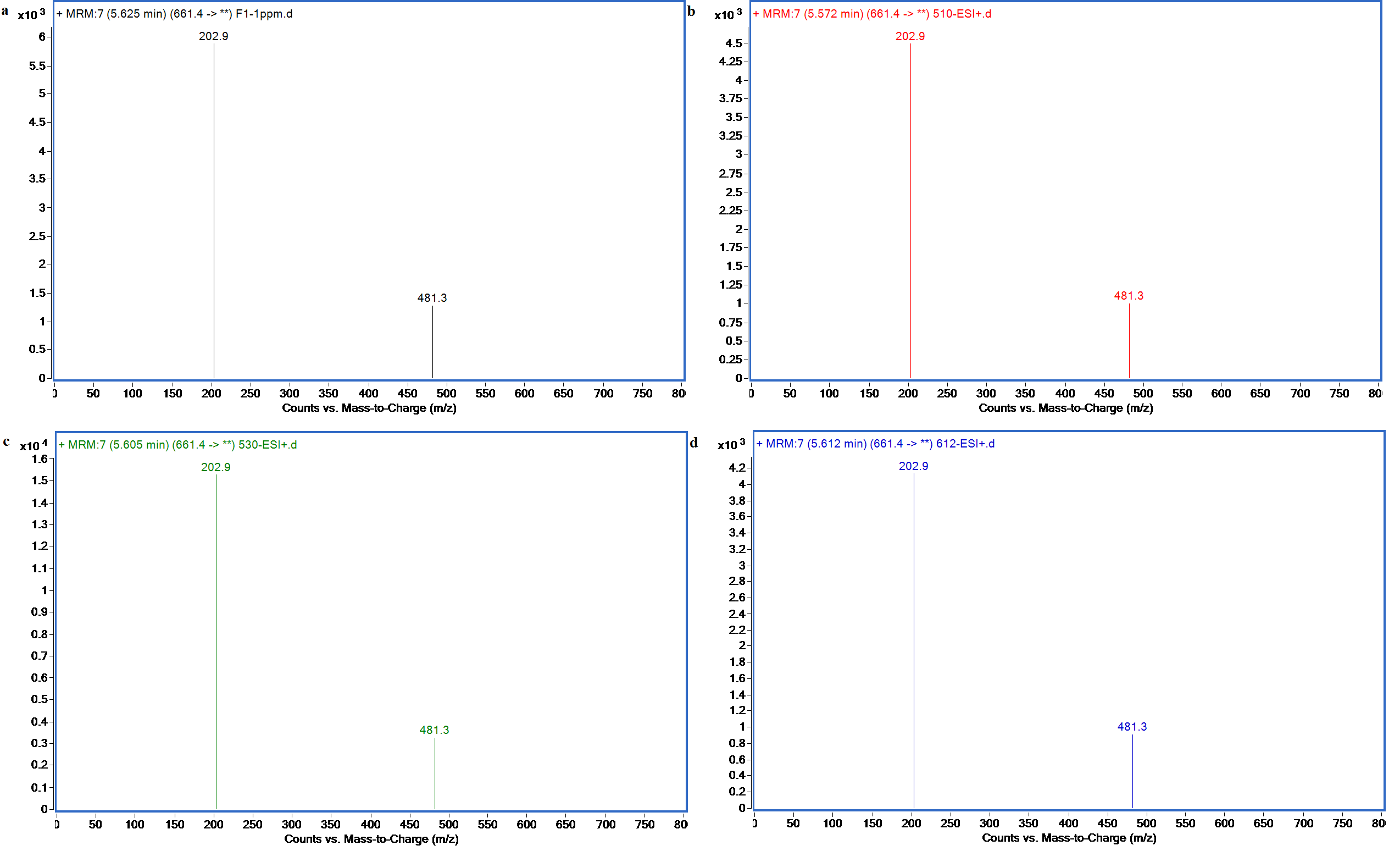

Supplement: Figure S23 — The mass spectrum of the F1 solution and the test sample solution. a. F1; b. the batch of 20110510; c. the batch of 20110530; d. the batch of 20110612. (TIF) [file pone.0078902.s023.tif]

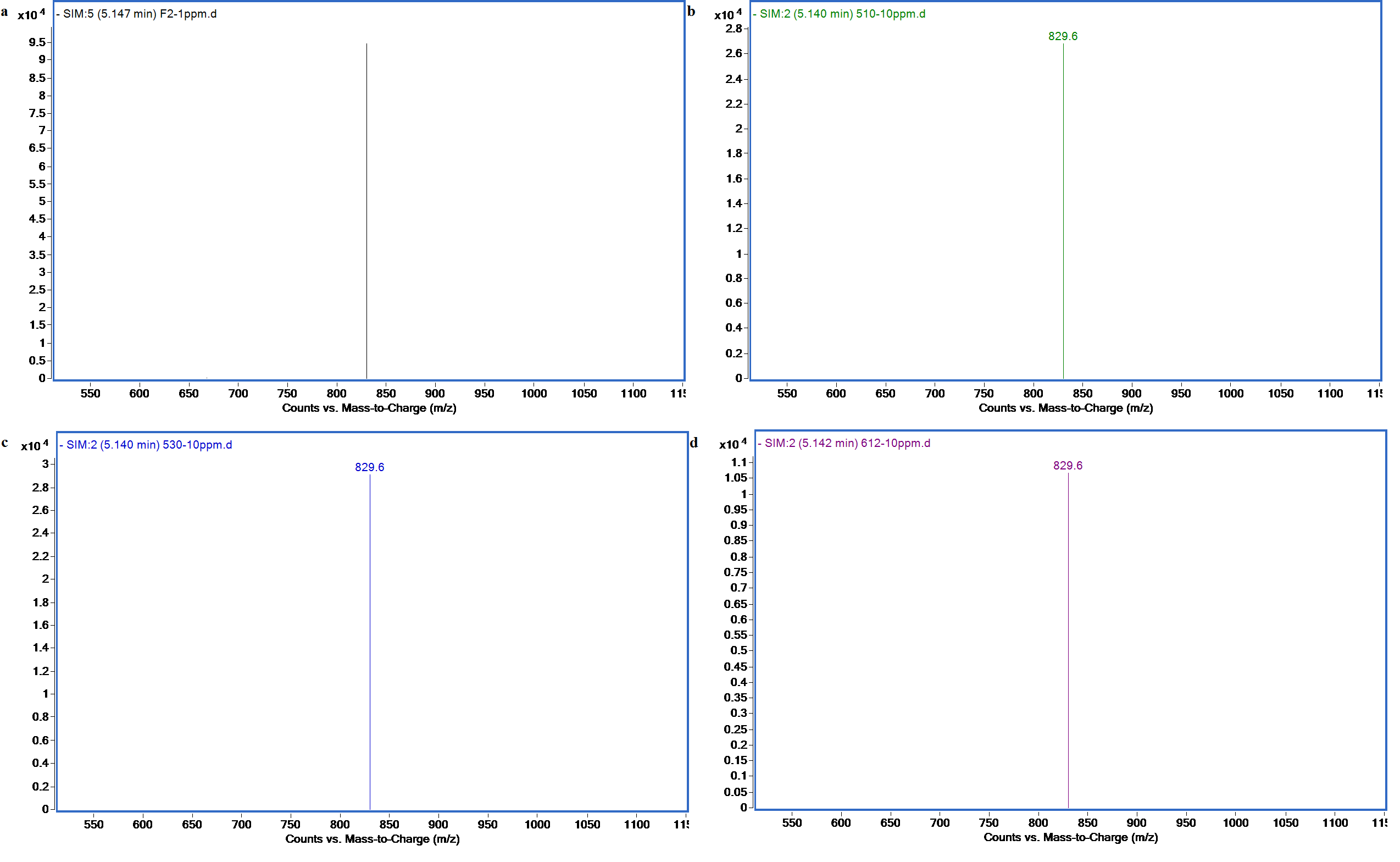

Supplement: Figure S24 — The mass spectrum of the F2 solution and the test sample solution. a. F2; b. the batch of 20110510; c. the batch of 20110530; d. the batch of 20110612. (TIF) [file pone.0078902.s024.tif]

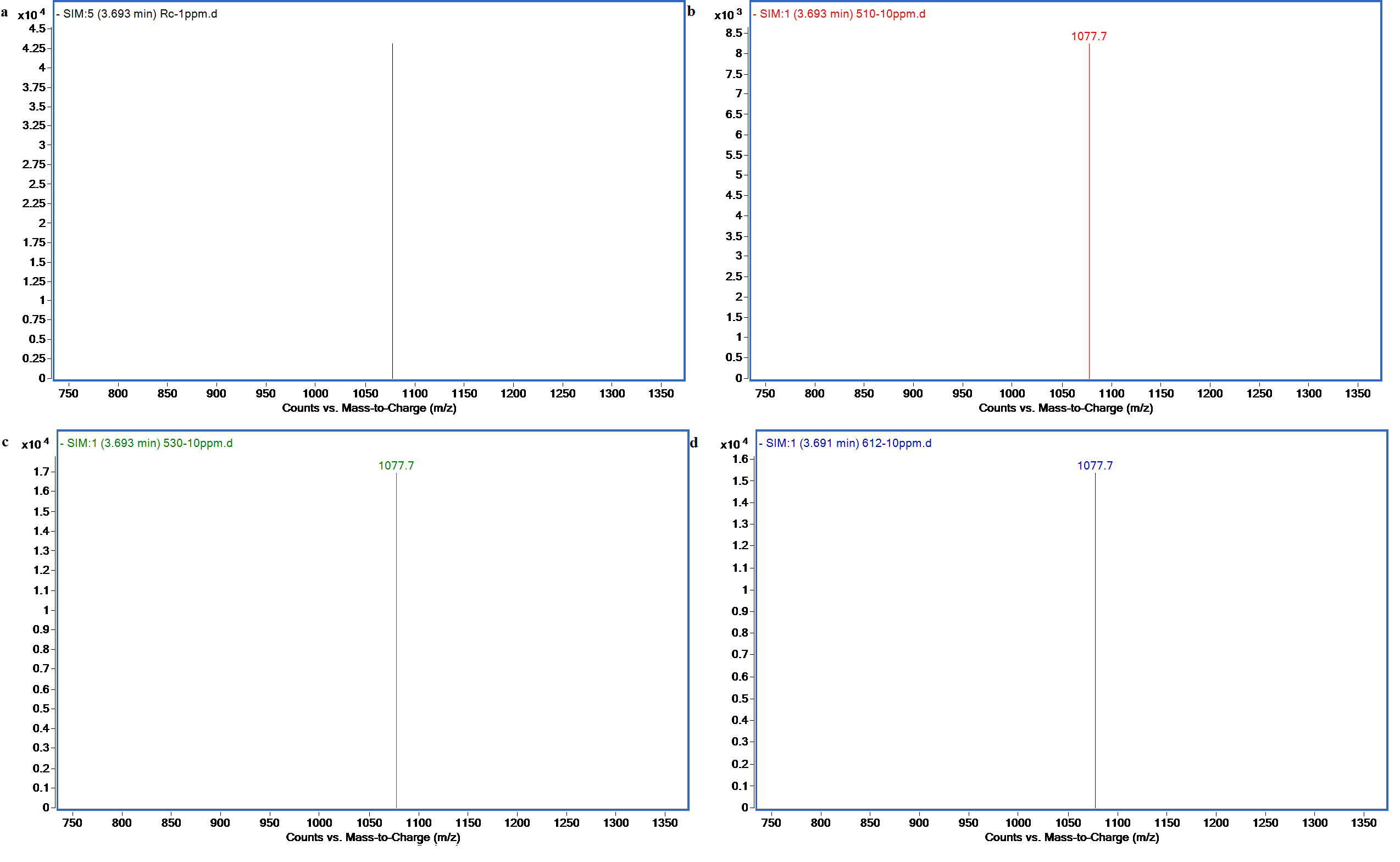

Supplement: Figure S25 — The mass spectrum of the Rc solution and the test sample solution. a. Rc; b. the batch of 20110510; c. the batch of 20110530; d. the batch of 20110612. (TIF) [file pone.0078902.s025.tif]

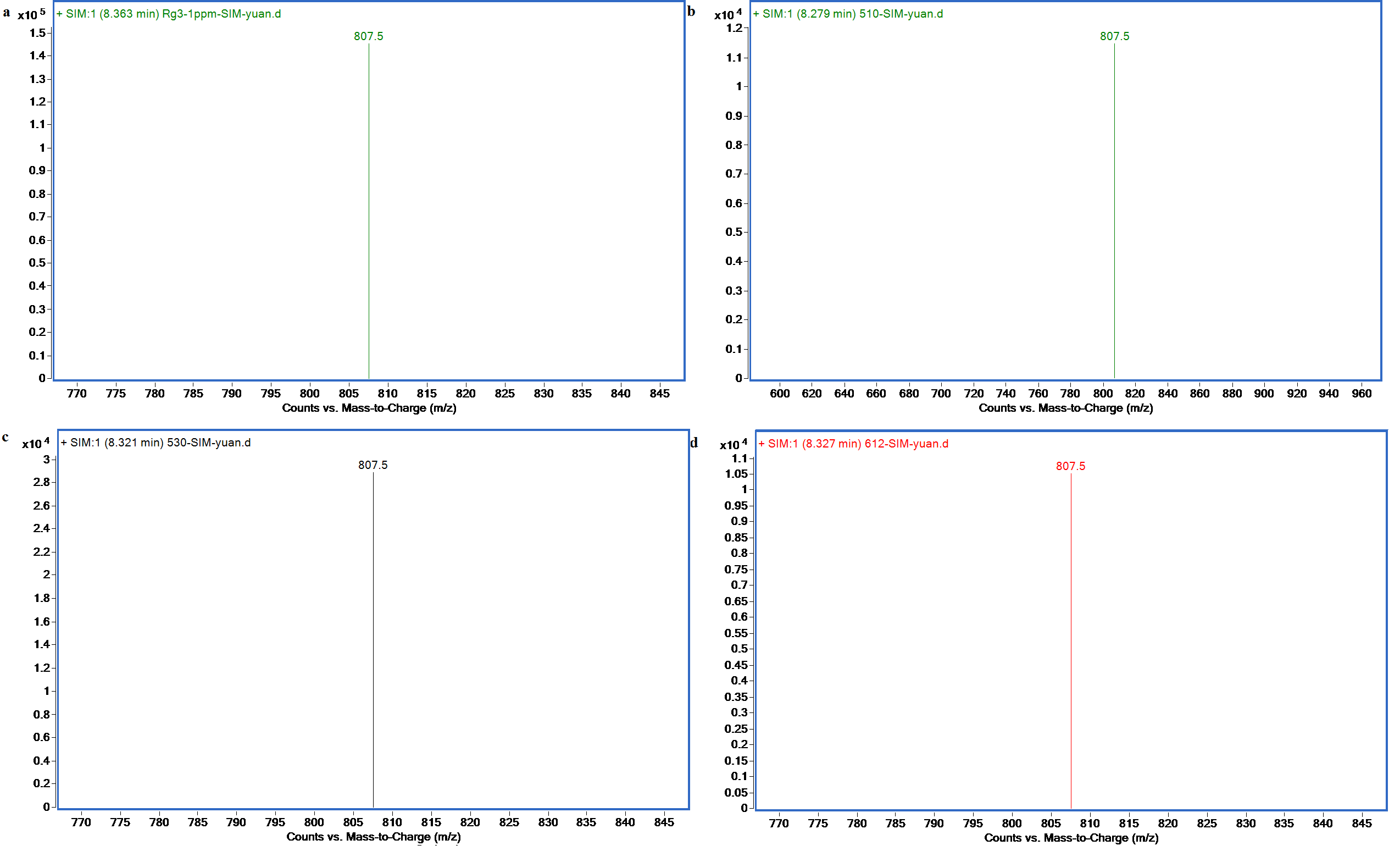

Supplement: Figure S26 — The mass spectrum of the Rg3 solution and the test sample solution. a. Rg3; b. the batch of 20110510; c. the batch of 20110530; d. the batch of 20110612. (TIF) [file pone.0078902.s026.tif]

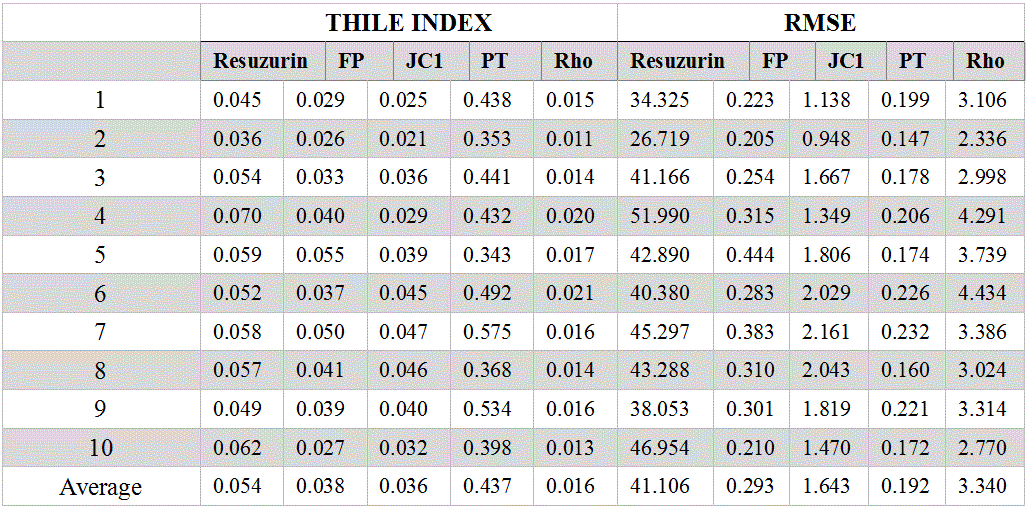

Supplement: Table S1 — THILE Index and RMSE for BPNN regression model. (TIF) [file pone.0078902.s028.tif]

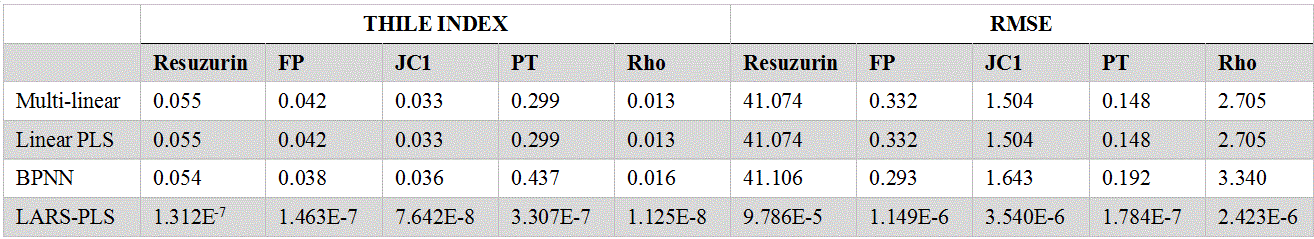

Supplement: Table S2 — Comparison of THILE Index and RMSE among the four models. (TIF) [file pone.0078902.s029.tif]

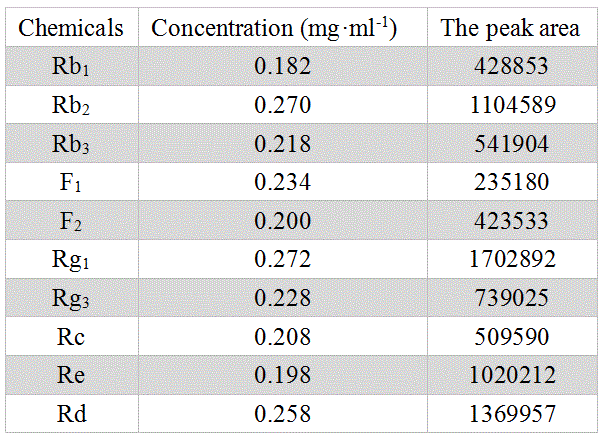

Supplement: Table S3 — The peak area of each ginsenosides standards. (TIF) [file pone.0078902.s030.tif]

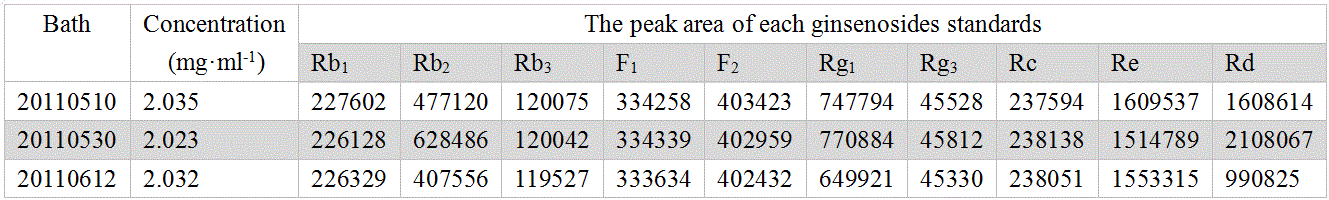

Supplement: Table S4 — The peak area of each ginsenosides standards in three batches. (TIF) [file pone.0078902.s031.tif]

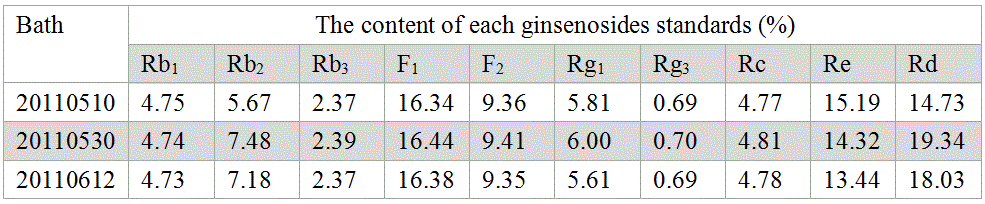

Supplement: Table S5 — The content of each ginsenosides standards in three batches. (TIF) [file pone.0078902.s032.tif]
