# Supplementary material for: An Effective Solution to Discover Synergistic Drugs for Anti-Cerebral Ischemia from Traditional Chinese Medicinal Formulae
Source: PLoS One. 2013 Nov 13;8(11):e78902. doi: 10.1371/journal.pone.0078902 (PMC3827340; doi:10.1371/journal.pone.0078902)
Supplement: Text S1 — Supporting Information Legends. (DOC) [file pone.0078902.s027.doc]

**Supporting Information:**

**Table S1.** THILE Index and RMSE for BPNN regression model

**Table S2.** Comparison of THILE Index and RMSE among the four models

**The preparation of standards solution and test sample solution**

Rb_1_, Rb_2_, Rb_3_, F_1_, F_2_, Rg_1_, Rg_3_, Rc, Re, Rd were made soluble in methanol (0.2 mg·ml^-1^).

Ginsenosides 20 mg was made soluble in 10 ml methanol, and filtrated. The filtration was obtained for the analysis.

**The content determination of ginsenosides.**

The liquid phase analysis was performed on an Shimadzu LC-20AT high performance liquid chromatography instrument (Kyoto, Japan), The analytes were separated on a Waters Sun Fire C18 column (5μm, 4.6×250mm) at a column temperature of 30℃. The mobile phase for elution was a gradient established between solvent A (acetonitrile) and solvent B (water) at a flow rate of 1mL·min^-1^. Baseline separation was achieved using a gradient starting from 0% A/ 100% B followed by a linear increase of A. Reaching 19% A from 0min to 15min; reaching 21% A from 15min to 20 min; reaching 26% A from 20 min to 25 min; reaching 27% A from 25 min to 45 min; reaching 32% A from 45 min to 60 min; reaching 43% A from 60 min to 75 min; reaching 60% A from 75 min to 90 min; reaching 100% A from 90 min to 100 min. The mass spectrum of the ginsenosides was performed on an Agilent 1200 rapid resolution liquid chromatography instrument (Santa Clara, CA, USA).

**Table S3** The peak area of each ginsenosides standards

**Table** **S4** The peak area of each ginsenosides standards in three batches.

**Table S5** The content of each ginsenosides standards in three batches.

**The liquid phase diagram of the standards solution and test sample solution were as follows:**

1. The liquid phase diagram of the test sample solution and the standards solution (Rb_2_, Rd, Re, Rg_1_)

**Figure S1.** The chromatogram of Rb_2_

**Figure S2.** The chromatogram of Rd

**Figure S3.** The chromatogram of Re

**Figure S4.** The chromatogram of Rg_1_

**Figure S5.** The chromatogram of test sample solution (20110510)

**Figure S6.** The chromatogram of test sample solution (20110530)

**Figure S7.** The chromatogram of test sample solution (20110612)

1. The liquid phase diagram of the test sample solution and the standards solution (Rb_1_, Rb_3_, Rc, F_1_, F_2_, Rg_3_)

**Figure S8.** The chromatogram of Rb_1_

**Figure S9.** The chromatogram of Rb_3_

**Figure S10.** The chromatogram of Rc

**Figure S11.** The chromatogram of F_1_

**Figure S12.** The chromatogram of F_2_

**Figure S13.** The chromatogram of Rg_3_

**Figure S14.** The chromatogram of test sample solution (20110510)

**Figure S15.** The chromatogram of test sample solution (20110530)

**Figure S16.** The chromatogram of test sample solution (20110612)

**The mass spectrum of the standards solution and test sample solution were as follows:**

**Figure S17.** The mass spectrum of the Rg_1_ solution and the test sample solution. a. Rg_1_; b. the batch of 20110510; c. the batch of 20110530; d. the batch of 20110612.

**Figure S18.** The mass spectrum of the Re solution and the test sample solution. a. Re; b. the batch of 20110510; c. the batch of 20110530; d. the batch of 20110612.

**Figure S19.** The mass spectrum of the Rd solution and the test sample solution. a. Rd; b. the batch of 20110510; c. the batch of 20110530; d. the batch of 20110612.

**Figure S20.** The mass spectrum of the Rb_1_ solution and the test sample solution. a. Rb_1_; b. the batch of 20110510; c. the batch of 20110530; d. the batch of 20110612.

**Figure S21.** The mass spectrum of the Rb_2_ solution and the test sample solution. a. Rb_2_; b. the batch of 20110510; c. the batch of 20110530; d. the batch of 20110612.

**Figure S22.** The mass spectrum of the Rb_3_ solution and the test sample solution. a. Rb_3_; b. the batch of 20110510; c. the batch of 20110530; d. the batch of 20110612.

**Figure S23.** The mass spectrum of the F_1_ solution and the test sample solution. a. F_1_; b. the batch of 20110510; c. the batch of 20110530; d. the batch of 20110612.

**Figure S24.** The mass spectrum of the F_2_ solution and the test sample solution. a. F_2_; b. the batch of 20110510; c. the batch of 20110530; d. the batch of 20110612.

**Figure S25.** The mass spectrum of the Rc solution and the test sample solution. a. Rc; b. the batch of 20110510; c. the batch of 20110530; d. the batch of 20110612.

**Figure S26.** The mass spectrum of the Rg_3_ solution and the test sample solution. a. Rg_3_; b. the batch of 20110510; c. the batch of 20110530; d. the batch of 20110612.
